# Integrating Electric Ambipolar Effect for High-Performance Zinc Bromide Batteries

**DOI:** 10.1007/s40820-024-01636-6

**Published:** 2025-02-13

**Authors:** Wenda Li, Hengyue Xu, Shanzhe Ke, Hongyi Zhang, Hao Chen, Gaijuan Guo, Xuanyi Xiong, Shiyao Zhang, Jianwei Fu, Chengbin Jing, Jiangong Cheng, Shaohua Liu

**Affiliations:** 1 State Key Laboratory of Precision Spectroscopy, Engineering Research Center of Nanophotonics and Advanced Instrument (Ministry of Education), School of Physics and Electronic Science, East China Normal University, Shanghai, 200241 People’s Republic of China; 2 Department of Chemistry, Tsinghua University, Haidian District, Beijing, 100084 People’s Republic of China; 3 School of Materials Science and Engineering, Xinjiang University, 666 Shengli Road, Urumqi City, 830046 People’s Republic of China; 4 School of Materials Science and Engineering, Zhengzhou University, 75 Daxue Road, Zhengzhou, 450052 People’s Republic of China; 5 State Key Lab of Transducer Technology, Shanghai Institute of Microsystem and Information Technology, Chinese Academy of Sciences, Shanghai, 200050 People’s Republic of China

**Keywords:** Electric ambipolar effect, Hydrated eutectic electrolyte, Electrostatic shielding, Zinc bromide batteries

## Abstract

The coupling of fast redox kinetics, high-energy density, and prolonged lifespan is a permanent aspiration for aqueous rechargeable zinc batteries, but which has been severely hampered by a narrow voltage range and suboptimal compatibility between the electrolytes and electrodes. Here, we unprecedentedly introduced an electric ambipolar effect for synergistic manipulation on Zn^2+^ ternary-hydrated eutectic electrolyte (ZTE) enabling high-performance Zn-Br_2_ batteries. The electric ambipolar effect motivates strong dipole interactions among hydrated perchlorates and bipolar ligands of L-carnitine (L-CN) and sulfamide, which reorganized primary cations solvation sheath in a manner of forming Zn[(L-CN)(SA)(H_2_O)_4_]^2+^ configuration and dynamically restricting desolvated H_2_O molecules, thus ensuring a broadened electrochemical window of 2.9 V coupled with high ionic conductivity. Noticeably, L-CN affords an electrostatic shielding effect and an *in situ* construction of organic–inorganic interphase, endowing oriented Zn anode plating/stripping reversibly for over 2400 h. Therefore, with the synergy of electro/nucleophilicity and exceptional compatibility, the ZTE electrolyte dynamically boosts the conversion redox of Zn-Br_2_ batteries in terms of high specific capacity and stable cycling performance. These findings open a window for designing electrolytes with synergetic chemical stability and compatibility toward advanced zinc-ion batteries.

**Supplementary Information:**

The online version contains supplementary material available at 10.1007/s40820-024-01636-6.

## Highlights

- Electric ambipolar effect motivates strong dipole interactions reorganized primary cations solvation sheath.
- Electrostatic shielding homogenized the distribution for nucleated Zn and facilized the orientated Zn deposition.
- The eutectic network of Zn^2+^ ternary hydrated eutectic electrolytes enables highly reversible and noteworthy Br_2_/Br^−^ reaction kinetics.

**Supplementary Information:**

The online version contains supplementary material available at 10.1007/s40820-024-01636-6.

## Graphical abstract

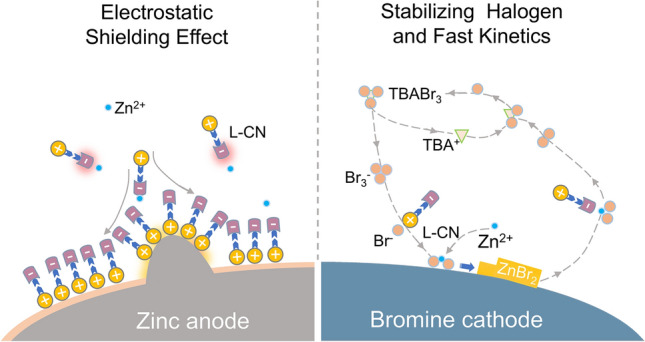

## Introduction

The integration of cost-effective, high-safety, and environmentally friendly battery systems with the electrical grid has proven beneficial, especially in ensuring continuous energy supply and addressing the intermittency challenges associated with renewable energy [1, 2]. Recently, rechargeable aqueous zinc-ion batteries have garnered considerable attention owing to their immense advantages in terms of exceptional safety, outstanding theoretical energy density, and economically sustainable raw materials [3–5]. Despite that, the practical implementation of aqueous zinc-ion batteries still remains hindered by several primary obstacles of thermodynamic and kinetic instability [6]. Specifically, conventional aqueous electrolytes frequently result in a shortened lifespan attributed to the uncontrolled formation of dendrites and the occurrence of competitive corrosion at interfaces on Zn anodes [7–9]. Moreover, the inherently narrow electrochemical stable window (ESW) of the electrolyte along with its poor compatibility with high-voltage cathodes prevents the achievement of high-energy density [10, 11]. Noticeably, as a representative of a high-energy density battery system, the two-electrons conversion reaction of a Zn-Br_2_ battery (ZBBs) based on the Br_2_/Br^−^ redox couple is appealing due to its exhibition of a high redox potential (1.7 V vs. Zn/Zn^2+^) alongside an impressive specific capacity of 335 mAh g^−1^ [12, 13]. Although ZBBs demonstrate intriguing advantages, the matching traditional aqueous electrolyte faces serious side reactions especially upon redox conversion under high voltage [14, 15]. To address these problems, it is crucial to design a compatible aqueous electrolytes system that realizes collaborative benefits in both the cathode and anode aspects, which contributes to fully demonstrating the potential.

Considering the aforementioned challenges linked to high-energy–density ZBBs, a thorough understanding and modulation of the cation solvation-interphase chemistry intimately associated with electrolytes become crucial for reversible Br_2_/Br^−^ conversion and stabilizing the Zn plating/stripping [16]. Although several strategies, such as component manipulation motivated water-in-salt electrolytes or introducing additives, to diminish the activity of the free water molecules and regulate cation solvation structure are proposed [17, 18], unfortunately, the unsatisfactory compatibility occurs when these materials are combined with aqueous electrolytes, primarily due to the dissolution of bromine species in the electrolyte solution. As a novel category of chemically stable and high tunability fluid materials, deep eutectic solvents (DES) are typically formed by blending Lewis (or Brønsted) acids and bases in virtue of robust donors/acceptors interactions (specifically, cation/dipole–dipole, and hydrogen bond) among the components [19]. Simultaneously, DESs are expected to have the ability to accommodate concentrated ionic species effectively, thereby contributing to widening the ESW and mitigating related side reactions for electrochemistry [20, 21]. Nevertheless, conventional liquid eutectic systems still suffer from large ion sizes and relatively high free volume, resulting in undesirable viscosity and limited ionic conductivity [22]. Currently, there remains a deficit in effective eutectic strategies to synergistically and comprehensively address these issues.

In this work, we introduce an unprecedented zwitterion electric ambipolar effect to ternary eutectic electrolytes by harmless dipolar ligands and low-cost hydrated salt for compatible high-energy density aqueous ZBBs. Notably, the synergetic modulating strategy of ternary eutectic electrolytes demonstrates remarkable universality, enabling the realization of nine distinct types of ternary eutectic electrolytes. Specifically, the systematical spectroscopic analyses associated with molecular dynamics simulations reveal the Zn^2+^ cations solvation shell in the form of the H_2_O-deficient Zn[(L-CN)(SA)(H_2_O)_4_]^2+^ six-coordinate configuration. Meanwhile, the ZTE affords an electrostatic shielding effect and then facilitates the *in situ* construction of an organic–inorganic hybrid solid electrolyte interface, which improved reversibility as well as satisfied Zn^2+^ kinetics, thereby enabling oriented Zn anode plating/stripping for over 2400 h. Crucially, the ZTE electrolyte can further minimize the energy barrier and enhance the efficiency of bromine conversion. The synergy enhancements of the anode and cathode aspects encompass a high capacity of 283.4 mAh g^–1^ at 0.5 A g^–1^, exceptional cycling stability (an activated capacity to 160.6 mAh g^–1^ with 85% retention over 3000 cycles), along with superior rate capabilities for static ZBBs. This work offers a new chance for advanced electrolytes by solvation modulation and interface regulation toward high-energy density aqueous ZBBs.

## Experimental Section

### Materials

Sulfamide (SA) and L-carnitine (L-CN) (99% purity) were purchased from Aladdin. Zinc trifluoromethylsulfonimide (Zn(OTf)_2_) and Zn(ClO_4_)_2_·6H_2_O were procured from Meryer. Tetrabutylammonium bromide (TBABr), sodium bromate (NaBrO_3_) and iodine powder were acquired from Adamas. Sigma-Aldrich supplies PVDF binders with an average molecular weight of approximately 1,000,000. N-methyl-2-pyrrolidone (NMP), with a purity of 99.9%, was obtained from Adamas. Analytical reagent-grade conductive carbon black, boasting a purity of 99%, was sourced from Aladdin. Carbon cloth, with a thickness of 0.02 mm, is obtained from CeTech. Co., LTD.

### Preparation of Electrolytes and Electrodes

#### Electrolytes Preparation

The ternary-hydrated eutectic electrolytes (ZTEs) based on zinc were formulated by blending L-carnitine (L-CN), sulfamide (SA), and Zn(ClO_4_)_2_·6H_2_O, following a heating process at 80 °C and subsequent cooling to room temperature. It is worth noting that the optimal molar ratio for this blend was determined to be 4:2:3 for L-CN, SA, and Zn(ClO_4_)_2_·6H_2_O, respectively. Additionally, a comparative reference electrolyte, 1 M Zn(ClO_4_)_2_ (ZW), was prepared by dissolving hydrated zinc perchlorate in water at ambient temperature.

#### ***Preparation of Containing Br***_***2***_*** Complex Electrodes***

For the preparation of Br_2_ electrodes, a slurry coating method on carbon cloth (CC) was adopted. Specifically, the Br_2_ complex electrode was formulated by blending 70 wt% TBABr_3_-solids, 10 wt% conductive carbon black as a conductivity enhancer, and 10 wt% PVDF as a binder in NMP solvent. This mixture was then deposited onto cleaned CC cut into 12-mm-diameter disks. The loading of the TBABr_3_-solids@C composite for electrochemical tests was set at 0.5 −1.0 mg cm^−2^. During the fabrication of Zn-Br_2_ pouch cells, the resultant slurry was coated onto a 5 cm × 8 cm piece of carbon cloth. The pouch cell is clamped securely with a glass plate to guarantee full electrode contact throughout the testing process. The coin-cells were constructed utilizing glass fiber as separator, with the Zn as the anode and TBABr_3_-solids@C composite serving as the cathode.

### Electrochemical Measurements

The electrochemical performance of Zn//Zn, Zn//Ti, and Zn-Br_2_ batteries was assessed using CR2032 coin-type cells at room temperature on a land battery test system. Linear polarization measurements were conducted in 1.0 M ZW and ZTE electrolytes using a three-electrode setup, comprising a bare Zn working electrode, a titanium foil counter electrode, and an Ag/AgCl reference electrode. Linear sweep voltammetry (LSV) measurements were conducted in nitrogen-saturated electrolytes, where platinum foil as the reference electrode and counter electrode, zinc foil as the working electrode. Galvanostatic cycling tests of the Zn-Br_2_ full battery were performed on a multichannel tester within a voltage range of 0.5–1.85 V. The specific capacities of the Zn-Br_2_ batteries were evaluated with respect to the mass of bromine. Impedance spectrum was analyzed using a CHI-760E instrument covering frequencies from 1 MHz to 0.1 Hz. Additionally, cyclic voltammetry (CV) tests were executed on the same system within a voltage window of 0.5 to 1.85 V with scan rates ranging from 0.1 to 0.5 mV s^−1^. The ionic conductivities (*σ*, mS cm^−1^) of the ZTE and ZW electrolytes were modeled by the following equation:

$$\sigma = d/RS$$

vwhere *d* represents the electrolyte thickness, *S* denotes the tested area of the electrolyte in the ion flow direction in square centimeters (cm^2^), and *R* stands for the bulk resistance (Ω), which equivalent to the ohmic resistance obtained through obtained by electrochemical impedance spectroscopy (EIS).

### Materials Characterization

The morphologies of Zn deposits on Zn metal anodes were characterized using a scanning electron microscopy (SEM, GeminiSEM-450), and EDS mapping was obtained at 10 kV. Raman spectra were acquired using an inVia Reflex system, utilizing an excitation wavelength of 532 nm. X-ray photoelectron spectroscopy (XPS) measurements were performed using a Thermo Fisher Scientific ESCALAB Xi + instrument with Al Kα radiation. Atomic force microscope (AFM) images were captured in tapping mode utilizing a Dimension icon AFM (Bruker). UV–vis spectroscopy was performed on a Lambda950 instrument. Fourier transform infrared (FTIR) was conducted on a Bruker Alpha spectrophotometer in reflection mode, spanning a wavenumber range of 4,000–400 cm^−1^. TOF–SIMS measurements were conducted with a PHI nano TOF II. A Bi^3+^ beam (30 keV, 100 × 100 μm^2^) was used as the primary beam to detect the samples, and sputtering with a Cs^+^ beam (2 kV, 140 nA, 300 × 300 μm^2^) was applied for depth profiling analysis. X-ray powder diffraction (XRD) patterns were collected on a Rying-AXS diffractometer (D8 ADVANCE) employing Cu–Kα radiation (λ = 1.5405 Å) at room temperature.

### Calculation Methods

The molecular dynamics (MD) simulations were performed to investigate the structural and dynamical properties of a ternary electrolyte system, composed of L-CN, SA, and Zn(ClO_4_)_2_·6H_2_O at a density of 1.7 g mL^−1^. The molar ratio of the components was set to 4:2:3, respectively. All simulations were conducted using the Desmond Molecular Dynamics package. The initial model of the electrolyte system was constructed by solvating the mixed solutes in a simulation box using packmol, ensuring the specified density and component ratios were achieved. The system was neutralized with appropriate counterions, and periodic boundary conditions were applied in all three dimensions. The MD simulations were carried out at 343 K using a Nose–Hoover thermostat and a Parrinello-Rahman barostat to maintain constant temperature and pressure, respectively. The OPLS-AA force field was employed to model the interactions between the atoms [23]. The system was first subjected to energy minimization to eliminate any bad contacts, followed by a 100 ps equilibration in the NVT ensemble to stabilize the temperature, and then a 100 ps equilibration in the NPT ensemble to stabilize the pressure. The production run was extended to 50 ns to adequately sample the configurational space and provide meaningful statistical averages. Trajectories were analyzed using the Desmond simulation event analysis tools. Radial distribution functions (RDFs) and coordination numbers were calculated to evaluate the solvation structure around the zinc ions. Transport properties such as diffusion coefficients were estimated from the mean squared displacement of the ions. The structural and dynamical data were supplemented with visual inspections of the trajectory using the VMD software [24]. The electrostatic potential was visualized using the Multiwfn software. Adsorption energy (*E*_a_) and corresponding differential charge density between eutectic electrolyte composition (SA, L-CN, and H_2_O) and Zn substrate were calculated with the generalized gradient approximation (GGA) in the form of the Perdew, Burke, and Ernzerhof (PBE) exchange–correlation functional, as implemented in the Dmol^3^ package:

$$E_{{\text{a}}} = E_{{{\text{total}}}} - E_{{\text{l}}} - E_{{{\text{Zn}}}}$$

The energy of isolated ligand and Zn matrix is defined as *E*_l_ and *E*_Zn_, and *E*_total_ represents whole system energy.

## Results and Discussion

### Design Concept and Physicochemical Properties of the Ternary-Hydrated Eutectic Electrolytes

**Fig. 1:**
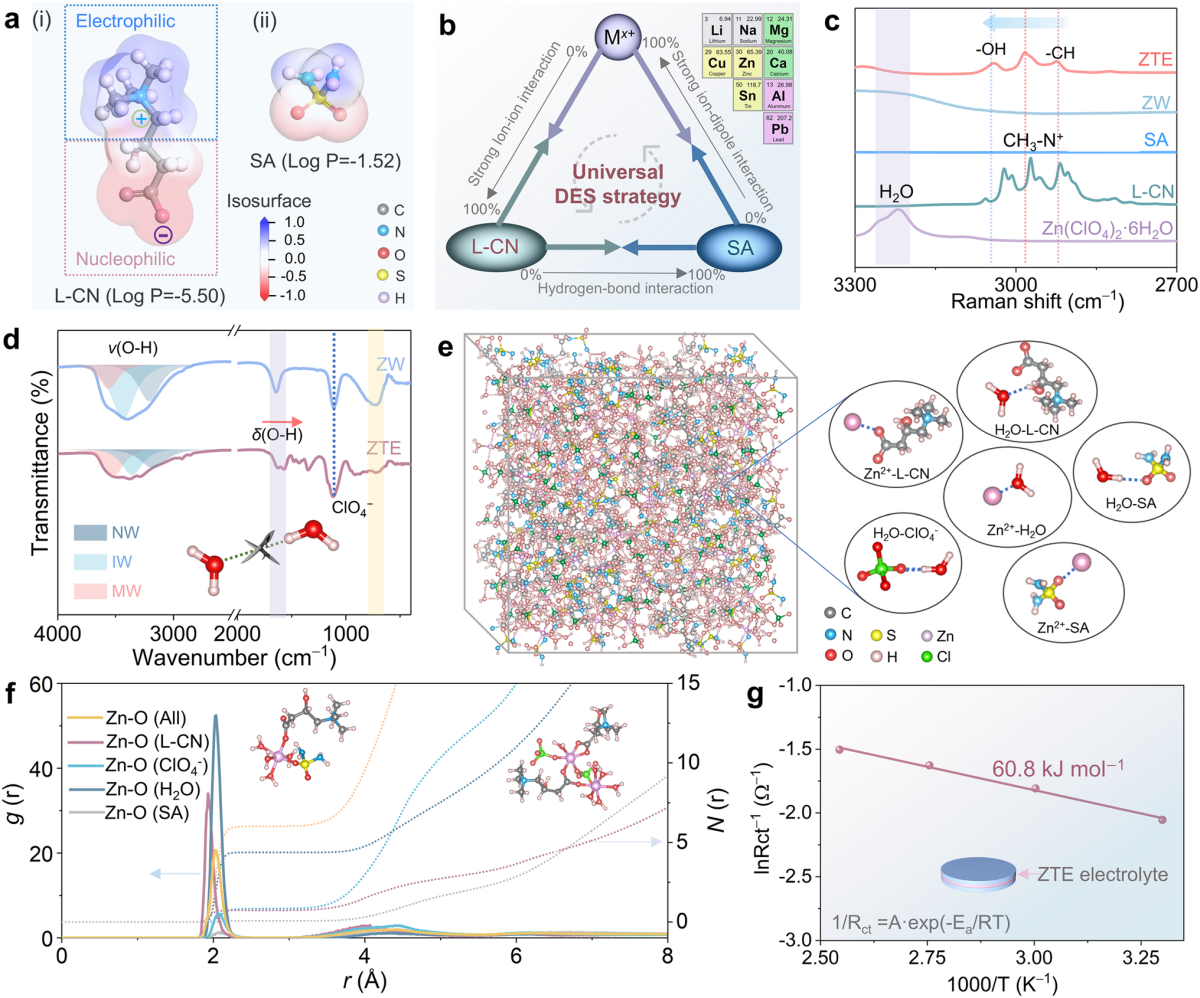
In-depth structural elucidation of ZTE and analysis of the Zn^2+^ coordinate configurations. **a** Electrostatic potential maps and corresponding log*P* values of L-CN and SA molecule, where *P* is the octanol–water partition coefficient serve to quantify the lipophilicity and hydrophilicity of components. **b** Schematic illustrating the universal design concept for hydrated deep eutectic electrolytes. **c** Raman spectra of L-CN, Zn(ClO_4_)_2_·6H_2_O, SA, ZW and ZTE. **d** The fitted FTIR spectra of ZW and ZTE electrolytes (Comparison of the areal ratios of multimer water (MW), intermediate water (IW), and network water (NW) in electrolytes based on the fitting of accumulated peaks). **e** Captured three-dimensional snapshots from molecular dynamics (MD) calculations and representative Zn^2+^-solvation structure of the ZTE. **f** The relevant radial distribution function (RDF) g(r) and coordination number N(r) pertaining to Zn–O interactions in MD simulation. **g** Arrhenius plots of inverse charge transfer resistance (R_ct_^−1^) values from 20 to 60 °C

L-carnitine (L-CN) as a green and widely available zwitterion is widely concerned for its biological activity in terms of energy metabolism, antifatigue, and antioxidants [25]. More interestingly, L-CN features both nucleophilic (COO^−^) and electrophilic ((CH_3_)_3_N^+^) groups, where the negatively charged COO^−^ in the zwitterion ligand exhibits a high affinity for metal cations through the chelation interaction that is responsible for its high aqueous solubility and severe hygroscopicity. It means that the carboxylic group can be theoretically incorporated into the aqueous electrolyte by utilizing salts containing electrophilic cation. Inspired by the above ambipolar characteristics of L-CN and eutectic chemistry insights, here, we propose a versatile strategy for the modulation of ternary-hydrated eutectic electrolytes by virtue of the electric ambipolar effect. The crucial physicochemical parameter, *log P*, is initially introduced as a metric to assess the lipophilic and hydrophilic tendencies of dipolar ligands and salt anions (Fig. 1a and Table S1). Specifically, the strong hydrophilicity (*log P* = −5.5) originated from the nucleophilic (COO^−^) group of the L-CN making it more preferred to bond with the H_2_O molecule by remodeling the hydrogen bonding network [26]. Moreover, in light of the structural features of a couple of hydrogen bond donors (high polarity S = O group) and a pair of hydrogen bond accepts (-NH_2_ moiety), along with similar amphiphilicity to ClO_4_^−^ and durability electrochemical stability (Fig. S1), the SA molecule was introduced into the hydrated eutectic electrolyte for further triggering the deep eutectic phenomenon by virtue of -NH_2_···O coordinate interaction with hydrated perchlorate. Benefitting from this unique design concept, a category of universal ternary-hydrated eutectic electrolytes can be modulated by blending M(ClO_4_)_*x*_·*y*H_2_O (where M = Zn^2+^, Li^+^, Na^+^, Cu^2+^, Pb^2+^, Mg^2+^, Ca^2+^, Sn^2+^, Al^3+^; *x* = 1, 2, 3; *y* = 6, 9) with L-CN and SA organic compounds, denotes the L-CN–M(ClO_4_)_*x*_·*y*H_2_O–SA system, featuring cost-effective, safe, and eco-friendly advantages (Figs. 1b and S2). Specifically, the manipulated zinc-ion ternary eutectic electrolyte at an optimal stoichiometric molar ratio (L-CN: SA: Zn salt = 4:2:3) exhibits a stable homogeneous liquid phase and highest ions conductivities at room temperature (Figs. S3 and S4). Furthermore, the H_2_O molecules stemming from hydrated salt play a crucial role in the establishment of hydrogen bond eutectic networks, thereby resulting in reduced viscosity and diminished solvating capacity.

The formation mechanism and molecular interactions among L-CN, SA, and Zn(ClO_4_)_2_·6H_2_O of the ZTE were systematically investigated by various spectroscopic analyses. Upon the formation of the eutectic liquid, there is a robust affinity interaction between the COO^–^ nucleophilic group and Zn^2+^ as evidenced by the blue shift of symmetric stretching vibrations of the COO^–^ group at around 1400 cm^–1^ (Figs. 1c and S5). Accordingly, the shifts in the H-C-H (1437 cm^–1^) and (CH_3_)_3_N^+^ (665 and 1688 cm^–1^) peaks indicate the formation of a small fraction of H-bonds between L-CN, SA, and H_2_O to build COO^–^…H or C-H…O interactions. It is noted that L-CN bearing polar oxygen-containing groups are capable of interacting with H_2_O to diminish the free water molecules. Both of these two bands shift to a higher frequency after forming the eutectic solution evidenced by the strengthening of intramolecular hydrogen bonds between organics and H_2_O molecules [27]. Furthermore, the reconstructed hydrogen bond networks are also verified by the FTIR and nuclear magnetic resonance spectra presented in Figs. 1d and S6, the combination of L-CN and SA leads to the destruction of the original water O–H networks, and then, the establishment of intermolecular H-bonds between the lone pair electrons present on the oxygen atom of bipolar ligands and isolated H_2_O [28]. In detail, the observed blueshift in the O–H vibrational band serves as an indicator of the augmentation in the strength of the O–H covalent bonds. This phenomenon underscores the superior electrochemical stability of MW compared to the other two types of water [29, 30]. Simultaneously, the ZTE electrolyte, which possesses a reduced content of NW and an increased proportion of MW, exhibits a diminished melting point along with enhanced electrochemical stability.

Theoretical simulations were conducted to further comprehensively identify the radial distribution functions and competitive coordination micro-environment between L-CN and SA with Zn^2+^ in the ZTE. The molecular dynamics simulation snapshots indicate that the H_2_O molecules in the primary Zn^2+^ coordination solvation sheath are partially replaced by L-CN and SA molecules (Figs. 1e and S7). It is observed that the L-CN and SA molecules interact with Zn^2+^ through COO^–^…Zn, SO_2_…Zn and OH…Zn ionic dipole/electric ambipolar effect, while the H_2_O molecule interacts with SA and ClO_4_^–^ anions through H_2_N-SO_2_…H–O and Cl-O…H–O hydrogen bond interaction patterns, respectively. Concretely, the Zn^2+^ is mainly coordinated by four H_2_O molecules, L-CN and SA molecules in the form of the Zn[(L-CN)(SA)(H_2_O)_4_]^2+^ six-coordinate configuration, and the cation charge is balanced by two molecules in the second solvation shell. Furthermore, the conclusion is further substantiated by the analysis of RDFs and coordination number (CN) distribution functions (Fig. 1f). Noticeably, owing to the presence of lone pair electrons in the COO^–^ group of the L-CN, a sharp peak of Zn…O (L-CN) pair emerges approximately at 1.8 Å (shorter than other coordination distances), which further reveals the strong electric ambipolar interaction and thus endowing L-CN to penetrate into the first solvated shell. Additionally, the coordination number N(r) of H_2_O in the primary Zn^2+^-solvation structural shell is approximately four. Therefore, the H_2_O molecules in the solvation sheath are efficiently de-cooperated with the central Zn ion by introducing L-CN and SA molecules based on the competitive solvation mechanism and then interacting with water molecules to establish a continuous hydrogen bonding network. Essentially, the reduced water content in the solvation sheath of Zn^2+^ and the weakly solvating electrolyte structure can accelerate the kinetics of Zn^2+^ plating/stripping. Additionally, the ion conductivities of electrolytes were examined at room temperature. Compared with the ZW electrolyte (4.6 × 10^–3^ S cm^−1^), the ZTE electrolyte presents a moderate ion conductivity of 3.2 × 10^–3^ S cm^−1^, which is attribute to intrinsic higher viscosity (246 cP). In addition, the activation energy (*E*_a_) for Zn^2+^ transfer was determined utilizing the Arrhenius equation (Figs. 1g and S8), revealing a reduction in *E*_a_ from 52.6 kJ mol^−1^ in the 1.0 M Zn(ClO_4_)_2_ electrolyte (ZW) to 60.8 kJ mol^−1^ in the ZTE electrolyte. Based on these, combined chemical thermodynamic analysis with density-functional theory (DFT) calculations revealed that the synergetic tailored ZTE electrolyte shows significant application potential in Zn-based batteries.

### High Reversible Zn Plating/Stripping in ZTE

**Fig. 2:**
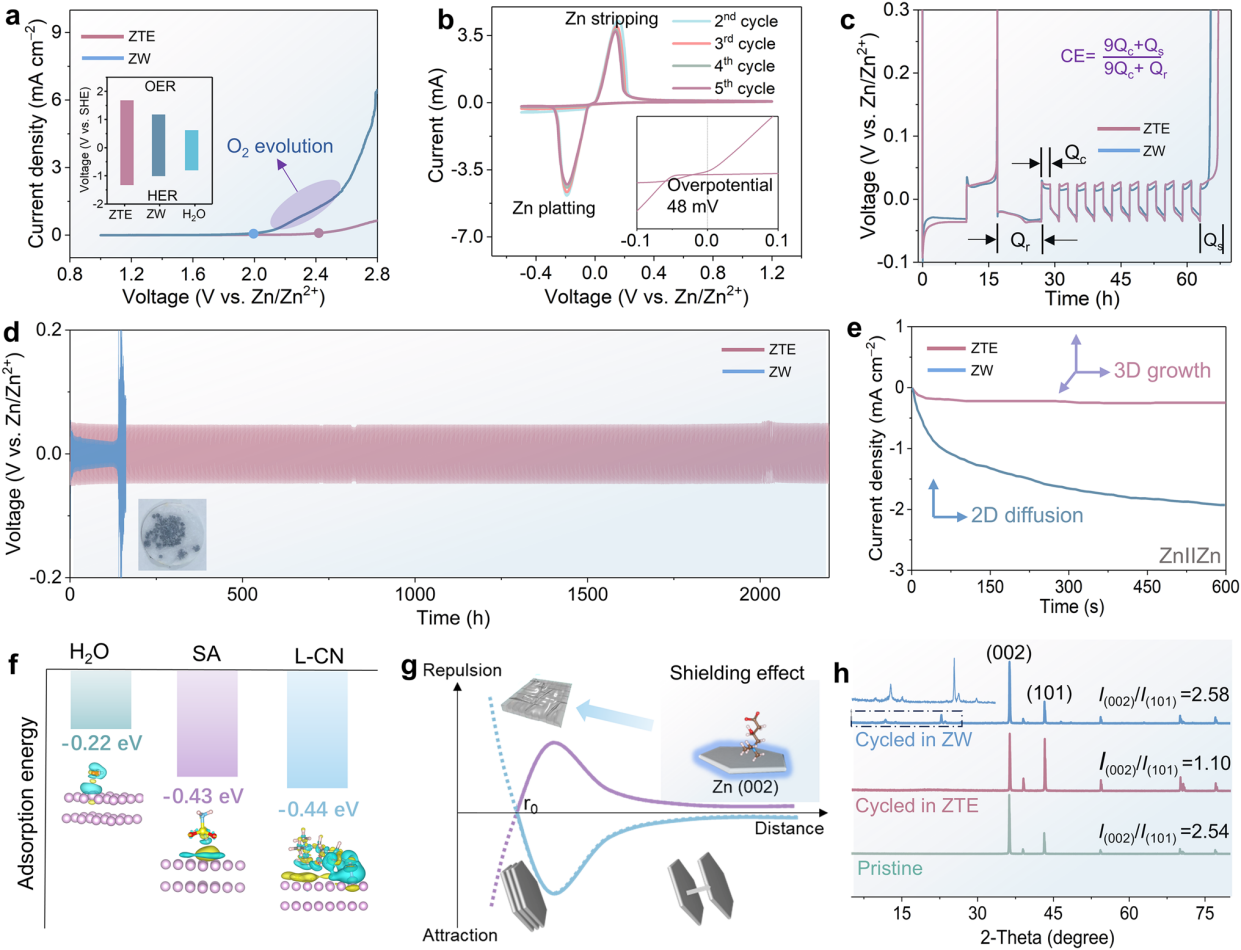
Electrochemical performance regarding Zn^2+^/Zn platting/stripping using ZTE and ZW electrolytes, respectively. **a** Plots of Tafel slopes for Zn electrode in the ZW and ZTE electrolytes. **b** Cyclic voltammetry curves of Zn//Ti batteries utilizing ZTE electrolyte at 0.5 mV s^−1^. **c** Profiles displaying voltage–time variations of Zn//Ti cells. **d** Voltage profiles of Zn//Zn symmetric cells undergoing galvanostatic plating/stripping in ZTE and ZW electrolytes with an area capacity of 0.5 mAh cm^−2^ (current density of 0.5 mA cm^−2^) for 2400 h (inset: the optical images of the Zn anode after 183 h cycled in ZW). **e** Chronoamperometric curves were recorded for the Zn anode during testing in the ZW and ZTE electrolytes at −150 mV. **f** Optimized geometric configuration and corresponding adsorption energies of H_2_O, L-CN, and SA molecular adsorbed onto the Zn (002) surfaces. **g** Schematic diagram of Zn platting in ZTE and ZW electrolytes. **h** XRD patterns of pristine Zn and Zn cycled in the electrolytes

To assess the availability of ZTE for the Zn anode, the Zn//Ti asymmetric cells were conducted to initially evaluate the ESW of ZTE by utilizing the linear sweep voltammetry (LSV) technique (Figs. 2a and S9). Benefiting from the minimized activity of the water molecules of hydrated Zn^2+^, the ZTE electrolyte delivers a remarkably wider ESW of 2.9 V (vs. Zn/Zn^2+^), significantly surpassing that of the baseline electrolyte (ZW) of 2.5 V (vs. Zn/Zn^2+^). More significantly, the asymmetric cell with ZTE electrolyte demonstrates enhanced redox behaviors (Fig. 2b). The potential of ZTE to facilitate the reversible Zn electrochemistry is fully explored through the application of cyclic voltammetry (CV) testing, which reveals a remarkably low initial voltage hysteresis of just 48 mV during Zn nucleation (as shown in the inset Fig. 2b), pointing to a minimal energy barrier associated with the phase transition between Zn^2+^ and Zn metal by using ZTE, thereby ultimately results in a reduction in the growth rate of Zn dendrites (Fig. S10) [31, 32]. To gain a more intuitive understanding of inhibition effect in dendritic growth and hydrogen evolution reactions during Zn deposition, we monitor the *in situ* optical micrograph along with quantifying the amount of hydrogen evolution. During *in situ* optical examinations of Zn deposition, observations revealed the gradual emergence of protrusions alongside bubbles on the Zn surface in ZW. Conversely, the Zn electrode within ZTE exhibited a consistently smooth and compact surface. (Fig. S11). Moreover, the effect was thoroughly assessed by evaluating the Coulombic efficiency (CE) of Zn//Ti asymmetric cells by using an Aurbach method [32]. As expected, the ZTE electrolyte endowed a higher CE of 90.6% than the ZW electrolyte (83%) (Figs. 2c and S12). Additionally, more compelling evidence for enhancing Zn plating/stripping by ZTE was acquired by testing the Zn symmetrical battery, the Zn//Zn batteries using ZTE demonstrated a stable plating/stripping process for 2400 h cycling without abnormal voltage fluctuation, significantly surpassing that of the ZW short-circuited within only 125 h (Fig. 2d), demonstrating significantly suppressed in side reactions and excellent reversibility. Furthermore, notable plating/stripping behaviors of Zn//Zn symmetric cells were also observed across various current densities ranging from 0.5 to 2.0 mA cm^−2^ (Fig. S13), proving impressive rate performances and rapid Zn^2+^ kinetics. Consequently, the ZTE is capable of attaining high integrated reversibility and favorable Zn^2+^ kinetics, surpassing the many of the other reported electrolyte systems.

To gain deeper insight into the mechanism underlying the growth of the deposited Zn layer, chronoamperometric measurements were conducted at a fixed over-potential of −150 mV. The ZTE electrolyte exhibits a reduced current response of −0.2 mA cm^−2^ compared to the ZW electrolyte (−2.5 mA cm^−2^), indicating that the ZTE electrolyte favors three-dimensional diffusion of Zn ions and promotes more uniform Zn deposition (Fig. 2e) [33]. Given that dendrite growth occurs precisely at the electrode–electrolyte interface, we leveraged DFT to analyze the adsorption modes and associated adsorption energies between various neutral ligands and the Zn substrate, aiming to elucidate the intrinsic mechanism behind (Fig. 2f). Significantly, there existed more robust interactions between organic ligands and the (002) crystal plane of the Zn substrate (−0.43 and −0.44 eV for SA and L-CN, respectively) than that of the H_2_O molecule (−0.22 eV). The preferential adsorption of L-CN would hinder the two-dimensional diffusion of Zn^2+^ for preventing further dendrite growth. Remarkably, a significant charge transfer is observed from the carboxyl group of the L-CN molecule to the Zn anode surface, indicating a rather strong chemical absorption to form the Zn–O bond.

Owing to the unique electric ambipolar effect exhibited by the L-CN ligand, the resulting solvated Zn^2+^ is potently adsorbed onto the solid/liquid interface under the action of the electric field. Specifically, the L-CN ligand preferred a vertical configuration with the electrophilic (CH_3_)_3_N^+^ group oriented on the Zn substrate (002), which results in a negatively charged shielding layer coming from the nucleophilic COO^−^ group on the Zn surface, thus significantly homogenizing the distribution for nucleated zinc and facilitated the directional deposition of Zn due to the electrostatic shielding effect. To facilitate a more intuitive description of the interactions between the various ligands and Zn substrate, the 3D models (Fig. 2g) depicting electron density statistics were constructed, the zincophilic functional groups (carboxyl and hydroxy groups) of L-CN reduce the desolvation energy barrier, thereby promoting the uniform platting of Zn in a vertical electric field. Therefore, compared to the traditional electrolytes, all these synergetic effects of the ZTE facilitate a more favorable and stable oriented Zn deposition along a thermodynamically stable (101) crystal plane without the side reactions. To verify the suspect occurrence at the Zn anode interface, a phase investigation was conducted on the deposited Zn anodes by employing XRD (Fig. 2h). All the peaks observed for the Zn anode after cycling in the ZTE electrolyte were exclusively attributed to Zn metal (PDF#00–004–0831), with no traces of any impurity phase detected. In contrast, additional diffraction peaks corresponding to Zn_4_ClO_4_(OH)_7_·*x*H_2_O (PDF#00–041–0715) were identified for the Zn anode cycled employing the ZW electrolyte, indicating a higher occurrence of side reactions compared to cycling in ZTE resulting from the hydrogen evolution reaction induced pH increase. Notably, the deposited Zn in the ZTE exhibits a distinct orientation favoring the (101) crystal plane, which is advantageous for inhibiting dendrite growth and corrosion-resistant due to the higher thermodynamic stability of the (101) crystal face.

### Understanding the Zn Deposit Structure and Interface Chemistry in ZTE

**Fig. 3:**
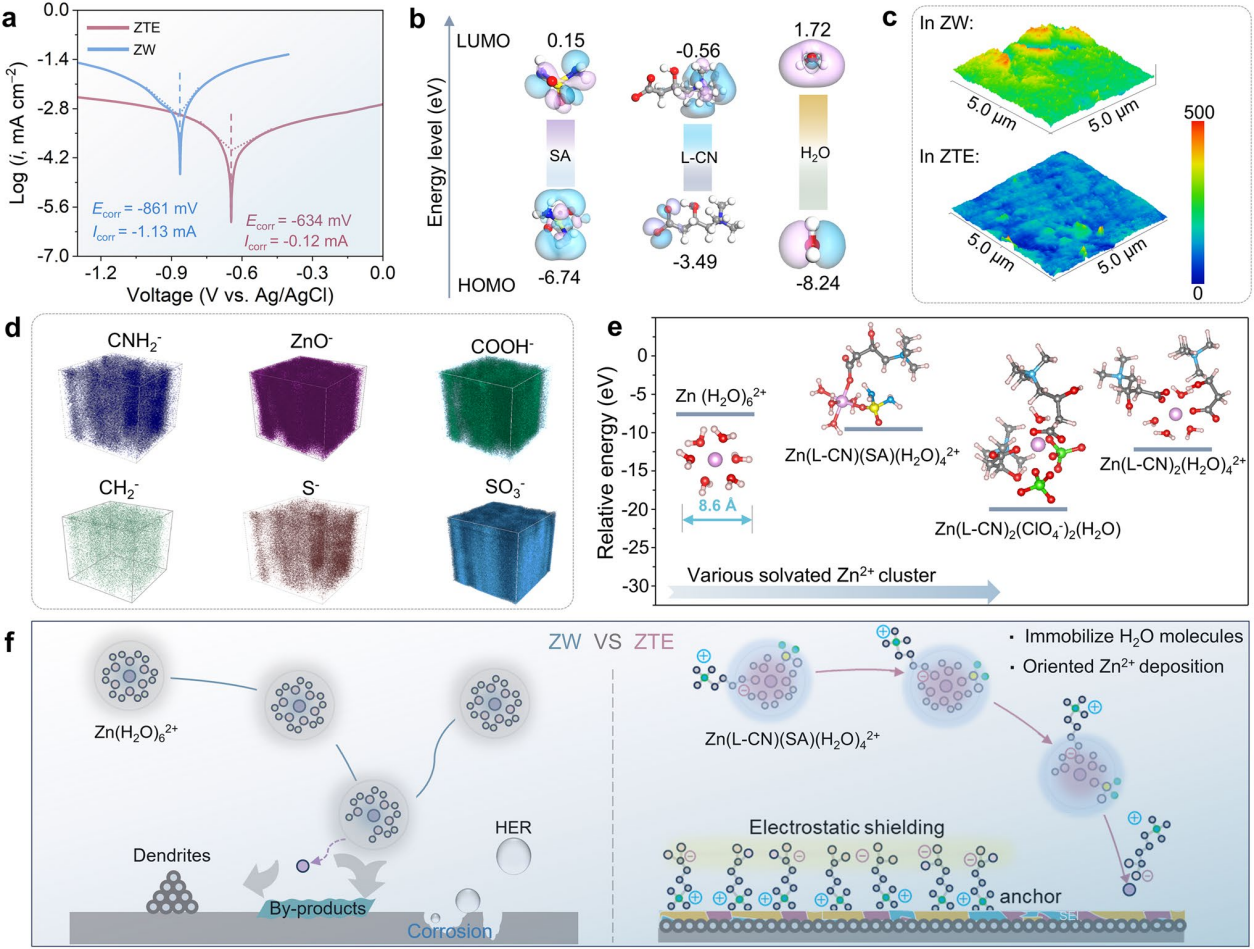
Comprehending the structure of Zn deposits and the interface chemistry. **a** Tafel plots of Zn electrode in the ZW and ZTE electrolytes. **b** LUMO and HOMO levels and corresponding isosurfaces of H_2_O, L-CN and SA molecules. **c** AFM 3D images of Zn foil after plating in ZW and ZTE electrolytes, respectively. **d** Three-dimensional visualization of Zn, S, O, and N elements distributions of SEI within the sputtered volumes obtained via the time-of-flight secondary-ion mass spectrometry (TOF–SIMS). **e** Dimensions and solvation energies of Zn^2+^ with different coordination structures. **f** Schematic diagrams comparing the Zn ion solvation structure diffusion of, desolvation, and adsorption deposition processes in ZW and ZTE electrolytes

To verify the superiority of the ZTE electrolyte for the Zn anode, we delved deeper into the intricate mechanism behind uniform Zn deposition through a combination of experimental investigation and theoretical calculations. Essentially, the uniform Zn plating is ascribed to the specialized solvation shells of electrolytes, which are oriented by ligands and exhibit affinity toward alkaline metal anodes [34]. Given the absence of free H_2_O in ZTE, in addition to the L-CN and SA ligands could be preferably absorbed onto the Zn surface in a coordinated manner. To verify the above conjecture, the anti-corrosion performance and corresponding Tafel curves of Zn electrodes are conducted (Fig. 3a). The Zn anode in ZTE displays a low corrosion potential (−0.634 V vs. Ag/AgCl) in comparison with the ZW electrolyte, indicating the exceptional ability to withstand corrosion behavior. In addition, the molecular orbital perspective elucidates the capacity for electron loss (Fig. 3b). The LUMO levels of L-CN and SA are situated below those of H_2_O, indicating suggesting that these compounds have a higher tendency to accept electrons, thereby hindering the decomposition of H_2_O. Additionally, their HOMO levels surpass those of H_2_O, potentially making it easier for them to donate electrons upon adsorption on the Zn surface and further facilitate the *in situ* formation of SEI interlayers [35]. The surface morphology and EDS mapping of Zn anode cycled in ZTE and ZW electrolytes was further scrutinized using an atomic force microscope (AFM) (Figs. 3c and S14). The topographic height image reveals a noticeably smoother Zn deposition in the ZTE electrolyte within a scanning area of 5 µm × 5 µm. The aforementioned phenomenon further indicates that ZTE can be preferentially adsorption onto the Zn anode, occupying the adsorption sites of Zn^2+^ and H_2_O molecules to form a protective layer (including SEI film and electrostatic shielding), thereby limiting the “tip effect” and parasitic side reactions.

In support of the aforementioned mechanism, we implemented a combination of techniques including time-of-flight secondary-ion mass spectrometry (TOF–SIMS), Raman analyses and XPS to experimentally probe the presence of the *in situ* formed interphase. The three-dimensional Zn anodes in the ZTE present the uniform SEI layer with homogeneous element distribution of Zn, C, O, and S (Fig. 3d). Moreover, organic components of CNH_2_, COO^−^ and favorable inorganic components of ZnCO_3_, ZnSO_3_, and ZnS are detected. The Raman together with XPS further evidenced the formation of SEI (Figs. S15 and S16). This hybrid SEI not only safeguards against water penetration but also guides Zn deposition, which results in the reduced kinetics of HER and dendrite growth, thereby boosting the reversibility of Zn plating/stripping [36]. Meanwhile, recognizing the close association between reaction kinetics and the desolvation capability of hydrated Zn ions, we also calculated the desolvation energies, taking into account the molecular geometries involved in the desolvation processes (Fig. 3e), a significant desolvation energy barrier of 0.83 eV is required for hydrated zinc in Zn(H_2_O)_6_^2+^ coordination complex. In comparison, the desolvation energy barriers are acceptably lower in the Zn[(L-CN)(SA)(H_2_O)_4_]^2+^ or Zn[(L-CN)_2_(H_2_O)_4_]^2+^ coordinating environments, indicating a more favorable promotion of the existence of the zwitterionic state during the desolvation process [37, 38]. Moreover, the expanded dimensions (12.5 Å) of Zn[(L-CN)(SA)(H_2_O)_4_]^2+^ can intensify the steric hindrance effect, resulting in a slower yet consistent diffusion process that suppresses the uncontrolled 2D diffusion (Fig. S17) [39]. Consequently, drawing upon the experimental analysis and theoretical models presented, Fig. 3f encapsulates the comprehensive mechanism for minimizing side reactions and maximizing the reversibility of Zn plating enabled by the ZTE electrolyte. In the Zn plating procedure, the robust interaction occurring between coordinated ligands (S = O and COO^−^ groups) of ZTE directs the plating along the (101) crystal plane [40]. Additionally, the distinctive solvated structures of ZTE remarkably minimized the protonation level of water molecules due to the hydrogen bonding locking effect, thus facilitating homogeneous nucleation and mitigating the development of the surface passivation layer.

### Redox Chemistry and Electrochemical Performance of Zn-Br_2_ Battery

**Fig. 4:**
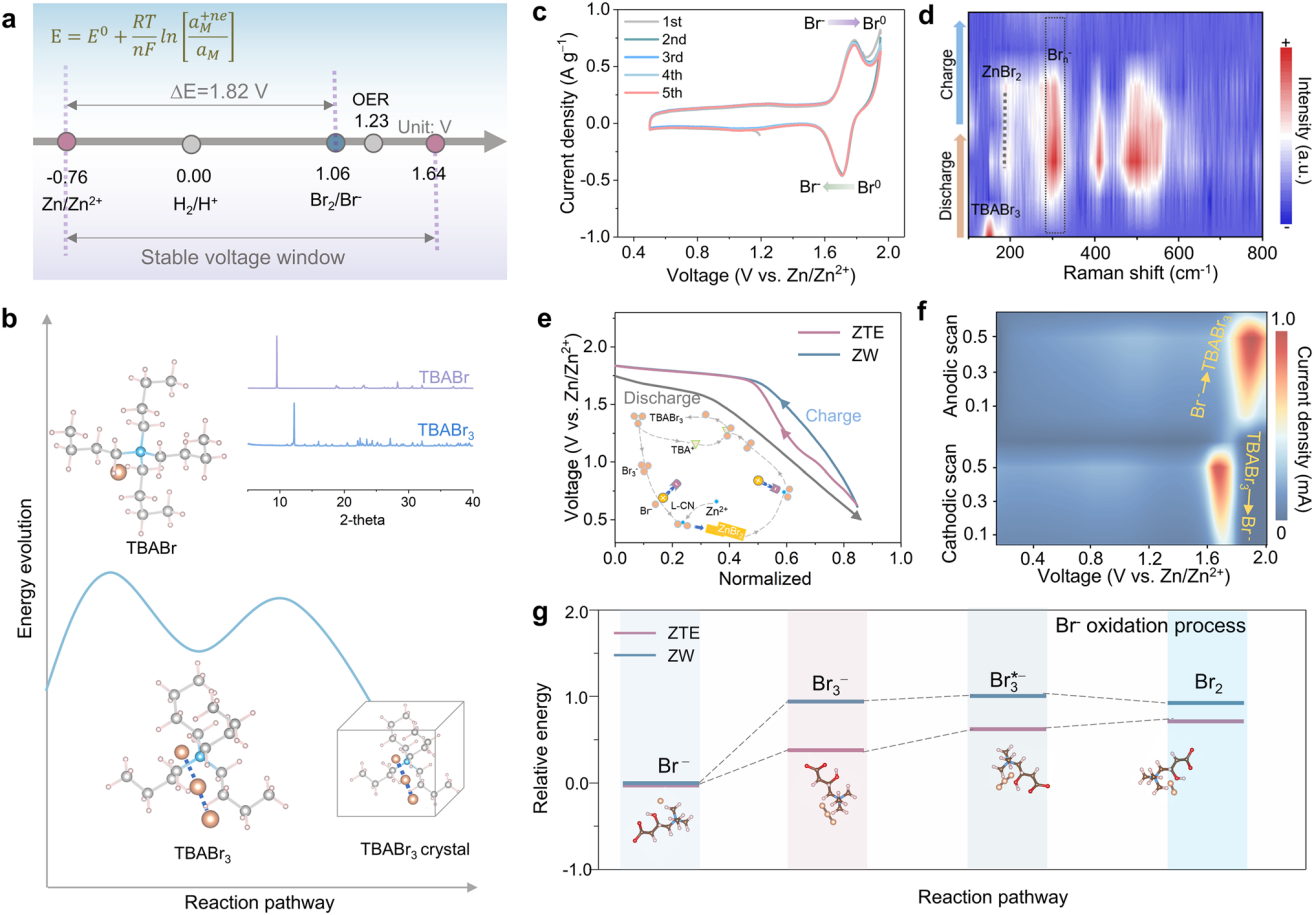
Redox chemistry and electrochemical performance of ZBBs. **a** Schematic of the stable working voltage window for ZTE electrolyte and Br_2_/Br^−^ redox chemistry. **b** Relaxed molecule structures and corresponding energy evolution process from the TBABr to TBABr_3_ (insert image corresponding to the XRD pattern of TBABr and TBABr_3_, respectively). The crystalline structure of TBABr_3_ chains has undergone testing and revealed a comparatively lower binding energy, indicating the potential for TBABr_3_ chains to form a dense network. **c** Typical CV curves of the Zn-Br_2_ battery using ZTE at a scan rate of 0.1 mV s^−1^. **d** ex situ Raman spectrum of ZBB using ZTE electrolyte. **e** A comparative analysis of the galvanostatic charge/discharge curves for ZBBs employing ZW and ZTE electrolytes from 0.5 to 1.95 V at 0.5 A g^−1^, respectively. **f** Contour plots of CV patterns of the Zn-Br_2_ battery using ZTE electrolytes at different scan rates. **g** Gibbs free energy diagrams of Br^−^ oxidation reaction in ZTE and ZW electrolytes, respectively. Where the * represents the active site

To validate the practicality and distinctive benefits of the ZTE electrolyte in Zn-based batteries with high-energy density, it was employed to be capable of the two-electron redox process ZBBs (Fig. 4a). Tetrabutylammonium bromide (TBABr) as an ionic compound consisting of the TBA^+^ cation and Br^−^ anion was selected as the Br_3_^−^ species efficient complexing agent to impede the cross-diffusion of Br_3_^−^ (Fig. S18) [41]. Notably, the dense network formed accompanied by the evolution of energy by TBABr_3_ chains is evidenced by the XRD pattern (inset of Fig. 4b), which could effectively inhibit bromine volatilization (Fig. 4b). A couple of typical redox peaks at 1.70/1.78 V are observed in the ZTE electrolyte (Fig. 4c), which is assigned to the Br_2_/Br^−^ conversion. While a reduction in the anodic cut-off current indicated the occurrence of an electrochemical side reaction, the remarkable coincidence of the CV curves in tandem with *ex situ* Raman and XPS spectrum (Figs. 4d and S19) further underscores the exceptional reversibility and stability in the redox conversion process. Additionally, we further systematically studied the behavior of the Br_2_/Br^−^ redox reaction. It is worthwhile mentioning that the ZTE electrolyte demonstrates huge advantages in terms of reducing the energy barrier for boosting bromine conversion, thus improving the energy efficiency of ZBBs (Fig. 4e). Impressively, attributing to the robust interaction between L-CN and iodine species, the ZTE electrolyte can facilitate the four-electron conversion of 2I^−^/I_2_/2I^+^ (Fig. S20**)**. Therefore, this discovery sheds light on the high-energy density exhibited by the four-electron Zn-I_2_ electrochemistry.

To uncover the underpinning of high-energy efficiency and rapid kinetics compelled by ZTE in the ZBBs, the reaction kinetics of Br_2_/Br^−^ can be further reflected by their cyclic voltammetry (CV) curves with different scan rates (Figs. 4f and S21). The contour plots of the CV patterns uncover a remarkable current response, especially in the region where ZnBr_2_ converts to Br_2_ species, indicating a rapid redox process. Besides, the two-electron reaction kinetics of Br_2_/Br^−^ in the ZTE electrolyte were systematically investigated according to the Tafel slope (*η*) ascertained from the CV curves (Fig. S22). Impressively, the Br_2_/Br^−^ redox process exhibited minimal *η* values of 64 mV dec^−1^, enabling highly reversible and noteworthy reaction kinetics. Moreover, we performed first-principles DFT calculations to clarify the spontaneous nature of the reaction and the enhancing role of ZTE in the Br^−^ the oxidation process, as evidenced by the corresponding Gibbs free energy diagram depicted in Fig. 4g. The small increased free energy (0.26 eV) of transition from Br^−^ to Br_3_^−^ indicates the ZTE facilitates the conversion, while the further transformation from Br_3_*^−^ to Br_2_ is blocked due to the increase in free energy (0.11 eV). Simultaneously, the integration of chemical thermodynamic analysis has elucidated a significant enhancement in the conversion of Br_3_^−^ as an intermediate during bromine oxidation reactions.

### Reversible Redox Chemistry of TBABr_3_ Enabled by ZTE

**Fig. 5:**
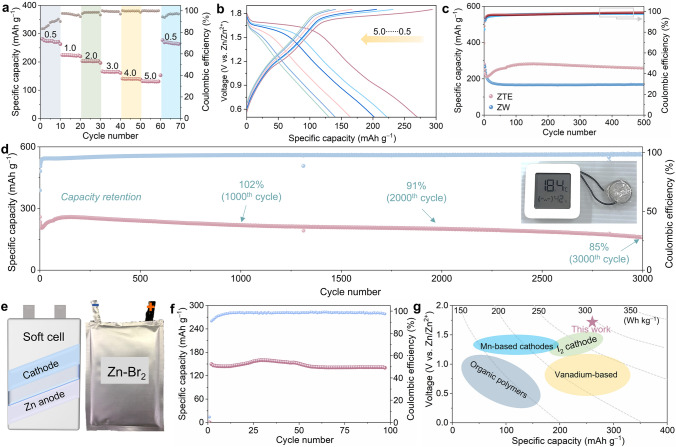
High-energy density Zn-Br_2_ conversion chemistries stabilized by ZTE. **a** Rate performance of ZBBs (the capacity is calculated based on the active material of Br_2_). **b** Typical charge–discharge profiles of ZBBs using ZTE electrolyte at various current densities. **c** Cycling performance and CE of the ZBBs with ZTE and ZW electrolytes at 0.5 A g^−1^. **d** Long-term cycling performance of the ZBBs at a current density of 0.5 A g^−1^ (inset: the optical image of the electronic thermometer powered by the ZBBs). **e** Configuration and the corresponding digital photograph of the Zn-Br_2_ pouch cell. **f** Cycling performance of a pouch cell at 1.0 A g^−1^. **g** A thorough comparison of the performance in terms of voltage and energy density, contrasted with prior findings on aqueous zinc-based batteries

In light of the fast kinetics and attractive Br_2_/Br^−^ redox chemistry performance by ZTE electrolyte, we investigated the rate performance and enduring cycle stability of the ZBBs equipped with the TBABr_3_ cathode to showcase their potential for practical energy storage applications. As shown in Figs. 5a and S23, the rate performance of ZBBs using ZTE electrolyte exhibits a noteworthy improvement compared to ZW. Specifically, the discharge capacities are 284.3, 212.5, 205.8, 158.6, 144.5, and 135.2 mAh g^−1^ at current densities of 0.5, 1.0, 2.0, 3.0, 4.0, and 5.0 A g^−1^, respectively. Moreover, upon reverting the current density to 0.5 A g^−1^, the average capacity could be recovered, indicating remarkable structural stability and a significant resilience toward rapid Zn^2+^ (de) intercalation in ZTE electrolyte. In addition, the corresponding charge/discharge curves of the ZBBs at various current densities (Fig. 5b). The charge/discharge plateaus are distinctly noticeable at a current density of 0.5 A g^−1^, aligning precisely with the oxidation/reduction peaks evident in CV curves. Furthermore, the carbon cloth demonstrates insignificant capacity during cycling (Fig. S24), suggesting that the battery's capacity is predominantly ascribed to the Br_2_ complex. Characterization of the cycling performance is pivotal to accessing the effectiveness of the ZTE electrolyte in inhibiting the polyiodide shuttle effect. At a rate of 0.5 A g^−1^ (Fig. 5c), the Zn-Br_2_ cell using ZW electrolyte demonstrates decay rapidly at its initial several cycles. In sharp contrast, the ZBBs with ZTE electrolyte deliver a high discharge capacity of 280 mAh g^−1^ and much better stability with a high-capacity retention over 500 cycles. It is worth mentioning that the initial decline in the capacity is probably attributed to the gradual activation of the electrode. Additionally, the EIS plots recorded during the pristine, second, and fifth cycles exhibit a noticeable reduction (Fig. S25), suggesting a transformation in the structural behavior of the bromine-rich cathode within the ZBBs over cycling.

More importantly, the long-term cycling performance of the ZBBs at high-current density was preferred (Fig. 5d), displaying a long-term cycle life with a high capacity of 160.6 mAh g^–1^ with a retention ratio of 85% even after 3000 cycles, suggesting the great promises of the ZTE electrolyte in Zn-Br_2_ cell for high-energy Zn batteries. One crucial criterion for the battery's suitability in large-scale energy storage applications is its electrochemical behavior at higher capacities. In this regard, we assembled the Zn-Br_2_ pouch cell with dimensions of 5 × 8 cm^2^ by adopting an alternating electrode stacking design. As depicted in Fig. 5e, the enlarged Zn-Br_2_ pouch battery can be stable in cycling for over 100 cycles with an average Coulombic efficiency of 96.5% (Fig. 5f). For comparison, we plotted the performance of our two-electron conversion ZBBs using the ZTE electrolyte in Fig. 5g alongside other aqueous systems (Table S2). The combination of the high discharge plateau of the Br_2_/Br^−^ redox couple and the remarkable specific capacity of the ZBBs, affording an impressive energy density of 292 Wh kg^−1^ (based on the bromine mass). This provides a compelling demonstration of the advantages of our system over conventional ZBBs, and positioning it uniquely among intercalation and conversion type electrodes for aqueous zinc-ion batteries.

## Conclusions

In summary, we proposed an innovative and effective approach in virtue of the electric ambipolar effect for designing ternary eutectic electrolytes toward compatible high-energy–density aqueous ZBBs. Notably, the synergistic tailored strategy for ternary-hydrated eutectic electrolytes exhibits significant universality capitalized on the electric ambipolar effect. The systematical spectroscopic analyses associated with molecular dynamics simulations revealed that the H_2_O-deficient Zn[(L-CN)(SA)(H_2_O)_4_]^2+^ six-coordinate configuration in the ZTE significantly broadens the ESW and orientates the Zn platting. Meanwhile, the ZTE affords an electrostatic shielding effect and *in situ* construction of an organic–inorganic hybrid solid electrolyte interface that facilitates the balances between improved reversibility and satisfactory Zn^2+^ kinetics, enabling oriented Zn anode plating/stripping for over 2400 h. Additionally, ZTE electrolyte also demonstrates huge advantages in terms of reducing the energy barrier for boosting bromine conversion. Concurrently, the synergistic improvements achieved in both the anode and cathode aspects boast a substantial capacity of 284.3 mAh g^–1^ at 0.5 A g^–1^, remarkable cycling stability that retains 85% of its activated capacity up to 160.6 mAh g^–1^ over 3000 cycles, and exceptional rate capabilities for static ZBBs. Our findings in eutectic chemistry engineering not only contribute to the advanced aqueous multivalent-ion batteries but also shed light on the rational design of electrolytes for the other energy storage applications.

## Supplementary Information

Below is the link to the electronic supplementary material.

Supplementary file1 (DOCX 7141 KB)
