# Supplementary material for: Integrating Electric Ambipolar Effect for High-Performance Zinc Bromide Batteries
Source: Nanomicro Lett. 2025 Feb 13;17:143. doi: 10.1007/s40820-024-01636-6 (PMC11825431; doi:10.1007/s40820-024-01636-6)
Supplement: Supplementary file 1 — Supplementary file1 (DOCX 7141 KB) [file 40820_2024_1636_MOESM1_ESM.docx]

Supporting Information for

**Integrating Electric Ambipolar Effect for High-Performance** **Zinc Bromide Batteries**

Wenda Li^1, †^, Hengyue Xu^2,^ ^†^, Shanzhe Ke^1, †^, Hongyi Zhang^1^, Hao Chen^1^, Gaijuan Guo^1,3^, Xuanyi Xiong^1^, Shiyao Zhang^1^, Jianwei Fu^4^, Chengbin Jing^1^, Jiangong Cheng^5^, Shaohua Liu^1^*

^1^ State Key Laboratory of Precision Spectroscopy; Engineering Research Center of Nanophotonics & Advanced Instrument (Ministry of Education), School of Physics and Electronic Science, East China Normal University, Shanghai 200241, P. R. China

^2^ Department of Chemistry, Tsinghua University, Haidian District, Beijing 100084, P. R. China

^3^ School of Materials Science and Engineering, Xinjiang University, 666 Shengli Road, Urumqi City, 830046, P. R. China

^4^ School of Materials Science and Engineering, Zhengzhou University,75 Daxue Road, Zhengzhou, 450052, P. R. China

^5^ State Key Lab of Transducer Technology Shanghai Institute of Microsystem and Information Technology, Chinese Academy of Sciences, Shanghai 200050, P. R. China

^†^ Wenda Li, Hengyue Xu, and Shanzhe Ke contributed equally to this work.

*Corresponding author. E-mail: [shliu@phy.ecnu.edu.cn](mailto:shliu@phy.ecnu.edu.cn) (Shaohua Liu)

**S1 Supplementary Experiments**

To prepare the TBABr_3_-based material, a gas-adsorption technique was employed. In a typical synthesis procedure, a solution named as A was prepared by dissolving 75 mmol of NaBrO_3_ in 25 mL of deionized (DI) water. Subsequently, 25 mL of 48% HBr was introduced to the mixture, yielding solution B, which was then uniformly dispersed through ultrasonic agitation. Meanwhile, 225 mmol of tetrabutylammonium bromide (C_16_H_36_NBr) was dissolved in 50 mL of DI water, forming solution C. Both solutions B and C were then combined in a beaker and magnetically stirred. Following stirring, the supernatant liquid was discarded, and the remaining the TBABr_3_-solids were dried.

**S2 Supplementary Calculations**

To derive the formula delineating the relationship between current density and potential on the anode, we make two fundamental assumptions:

(1) Simultaneously occurring on the anode are solely two electrode reactions: the dissolution reaction and the depolarization reaction. Both reactions are governed by activation polarization, with their rates being primarily controlled by this factor. Furthermore, the mass transfer process within the solution proceeds extremely rapidly, rendering concentration polarization negligible.

(2) The corrosion potential lies significantly apart from the equilibrium electrode potential of both aforementioned reactions. This ensures that both reactions are under strong polarization conditions, thereby allowing us to disregard the inverse processes of these reactions.

Given these assumptions, the kinetics of each electrode reaction can be adequately described using the Butler-Volmer (B-V) formula:

*i*_a,__I_ = $i_{I}^{0}exp\frac{2.3\left( E-E_{e, I} \right)}{b_{a,I}}$ (S1)

*i*_c,II_ = $i_{II}^{0}exp\frac{2.3\left( E-E_{e, II} \right)}{b_{c,II}}$ (S2)

Here, the subscript I denotes the electrode reaction associated with the metal anode dissolution in the anode direction, whereas the subscript II signifies the electrode reaction pertaining to the cathodic reduction of the depolarizer in the cathode direction. Specifically, *i*_a,I​_ represents the dissolution rate of zinc, and *i*_c,II​_ represents the evolution rate of hydrogen. The symbols *b*_a,I​_ and *b*_c,II_​ are defined as 2.3*RT*/*β*_I_​*n*_I_​*F* and 2.3*RT*/*α*_II_​*n*_II_​*F* respectively, where *R* is the universal gas constant, *T* is the absolute temperature, *F* is Faraday's constant, *n*_I​_ and *n*_II​_ are the number of electrons transferred in each reaction, and *β*_I​_ and *α*_II_​ are the symmetry factors.

When the corroded metal electrode is at its self-corrosion potential, the externally measured current is zero. The potential of this corroded metal electrode, known as the corrosion potential *Ecorr*​, satisfies the equation given in formula S3.:

𝑖_𝑎,I_ = 𝑖_𝑐,II_ = 𝑖_𝑐𝑜𝑟𝑟_ (S3)

*i*_c,II_ = $i_{I}^{0}exp\frac{2.3\left( E-E_{e, I} \right)}{b_{a,I}}$ = $i_{II}^{0}exp[-\frac{2.3\left( E-E_{e, II} \right)}{b_{c,II}}]$*= i*_𝑐𝑜𝑟𝑟_  (S4)

In other words, the absolute value of the current density of the anode reaction on the metal electrode is equivalent to that of the cathode reaction, and both are equal to the average corrosion current density of the metal electrode. The externally measured anodic polarization current density *i_A_*​ and cathodic polarization current density *i_C_*_​_of the corroded metal electrode can be expressed as follows:

*i*_A_ = *i*_𝑎,I_ − *i*_𝑐,II_ (S5)

*i*_C_ = *i*_𝑐,II_ − *i*_𝑎,I_ (S6)

Formula S1 and S2 are substituted into formula S5 and S6, then:

𝑖_𝐴_ = $i_{I}^{0}exp\frac{2.3\left( E-E_{e, I} \right)}{b_{a,I}}$*-* $i_{II}^{0}exp[-\frac{2.3\left( E-E_{e, II} \right)}{b_{c,II}}]$ (S7)

𝑖_C_ = $i_{II}^{0}exp[-\frac{2.3\left( E-E_{e, II} \right)}{b_{c,II}}]$*-* $i_{I}^{0}exp\frac{2.3\left( E-E_{e, I} \right)}{b_{a,I}}$ (S8)

Formula S4 is substituted into formula S7 and S8, then:

𝑖_𝐴_ = *i*_corr_{$exp\frac{2.3\left( E-E_{e, I} \right)}{b_{a,I}}$*-* $exp[-\frac{2.3\left( E-E_{e, II} \right)}{b_{c,II}}]\}$ (S9)

𝑖_C_ = *i*_corr_{$exp[-\frac{2.3\left( E-E_{e, II} \right)}{b_{c,II}}]$*-* $exp\frac{2.3\left( E-E_{e, I} \right)}{b_{a,I}}\}$ (S10)

where, 𝐸−𝐸_𝑐𝑜𝑟𝑟_=∆𝐸 is called the polarization value of corroded metal, and formulas S9 and S10 are the relationship of anode polarization curve and cathode polarization curve under electrochemical polarization, which is also called the basic formula of metal corrosion kinetics. If the positive and negative current signs are considered, the formulas S9 and S10 can be unified into the following formula:

𝑖 = *i*_corr_{$\exp\frac{2.3\Delta E}{b_{a,I}}$*-* $exp[-\frac{2.3\Delta E}{b_{c,II}}]\}$ (S11)

When the polarization Δ*E*=0, *i*=0, indicating that the corrosion system is in an open-circuit state. When the polarization Δ*E*>0, *i*>0, the corroded metal electrode undergoes anodic polarization. Conversely, when the polarization Δ*E*<0, *i*<0, the corroded metal electrode undergoes cathodic polarization. If the cathodic reaction rate of the corrosion process is influenced by the diffusion of the depolarizer in the electrolyte, additional considerations are necessary to supplement the formula. In the presence of concentration polarization, the relationship between the absolute value of the current density and the electrode potential is described as follows:

$\left| i_{C} \right|=$(1-$\frac{\left| i_{C} \right|}{i_{L}}$) $i_{C}^{0}$exp[-$\frac{2.3(E-E_{c, e})}{b_{c}}$] (S12)

where 𝑖_𝐿_ is the limiting diffusion current density. Substituting the relationship of |𝑖_𝐶_|= 𝑖_𝑐𝑜𝑟𝑟_ when 𝐸 = 𝐸_𝑐𝑜𝑟𝑟_ into equation S12, then:

|𝑖_𝐶_|=$\frac{i_{\mathrm{corr}}\exp\frac{2.3\Delta E}{b_{c}}}{1-\frac{i_{\mathrm{corr}}}{i_{L}}[1-exp(-\frac{2.3\Delta E}{b_{c}})]}$ (S13)

Thus, the polarization curve equation of the corroded metal electrode is obtained:

𝑖 = *i*_corr_{$\exp\frac{2.3\Delta E}{b_{a,I}}$*-*$\frac{i_{\mathrm{corr}}\exp\frac{2.3\Delta E}{b_{c}}}{1-\frac{i_{\mathrm{corr}}}{i_{L}}[1-exp(-\frac{2.3\Delta E}{b_{c}})]}\}$ (S14)

In certain circumstances, when the corrosion process is governed by the cathodic diffusion process, the corrosion current density becomes equivalent to the absolute value of the diffusion current density associated with the cathodic reaction, denoted as *i_corr_* ​≈ *i_L_*​. In such a scenario, this relationship can be derived from equation S14.

𝑖 = *i*_corr_$(\exp\frac{2.3\Delta E}{b_{a}}$*-*$1)$ (S15)

Therefore, formula S15 represents the polarization curve controlled by concentration polarization.

**S3 Supplementary Figures**


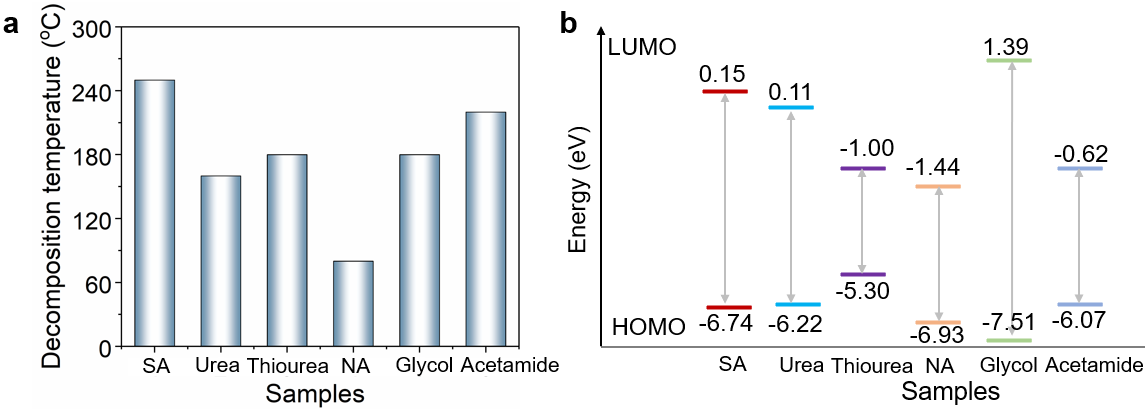


**Fig. S1** (**a**) The decomposition temperature and (**b**) LUMO and HOMO energy levels of the SA, urea, thiourea, NA (niacinamide), glycol and acetamide, respectively.

**Note**: The SA demonstrates the highest decomposition temperature and considerable HOMO-LUMO energy gap among selected alternatives, emphasizing the unique benefits of SA in thermostability durability and electrochemical stability.


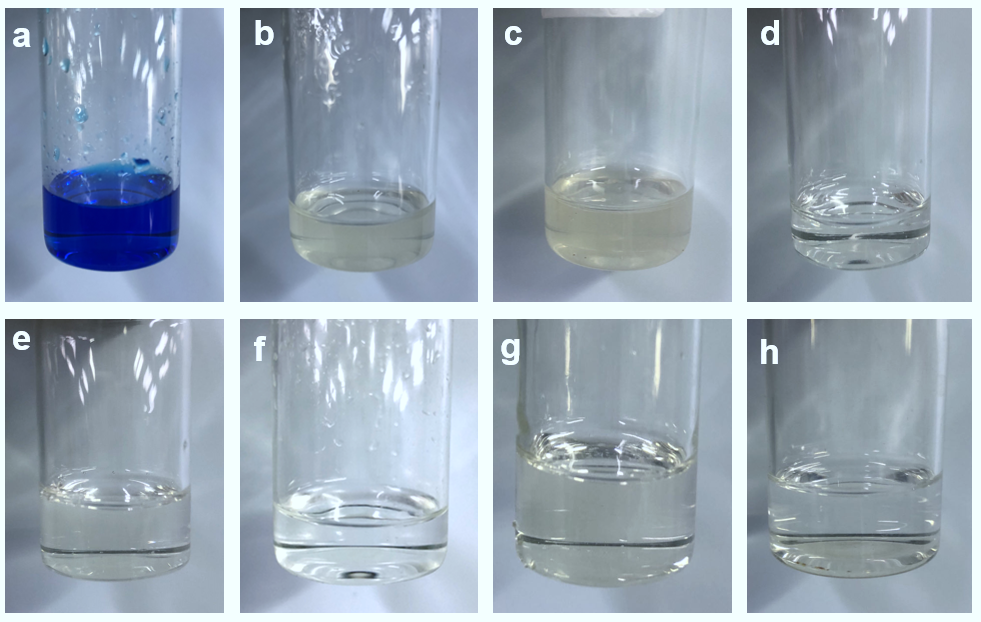


**Fig. S2** The optical photographs of the preparation various of hydrated eutectic electrolytes. (**a**) L-CN–Cu(ClO_4_)_2_·6H_2_O–SA, (**b**) L-CN–LiClO_4_·6H_2_O–SA, (**c**) L-CN–NaClO_4_·6H_2_O–SA, (**d**) L-CN–Mg(ClO_4_)_2_·6H_2_O–SA, (**e**) L-CN–Sn(ClO_4_)_2_·9H_2_O–SA, (**f**) L-CN–Pb(ClO_4_)_2_·9H_2_O–SA, (**g**) L-CN–Ca(ClO_4_)_2_·6H_2_O–SA and (**h**) L-CN–Al(ClO_4_)_3_·9H_2_O–SA

**Note**: The manipulated hydrated eutectic electrolytes exhibit a homogeneous liquid phase at room temperature at a different optimal stoichiometric molar ratio. With a long period of resting, some of them undergo a weak transition to the solid phase.


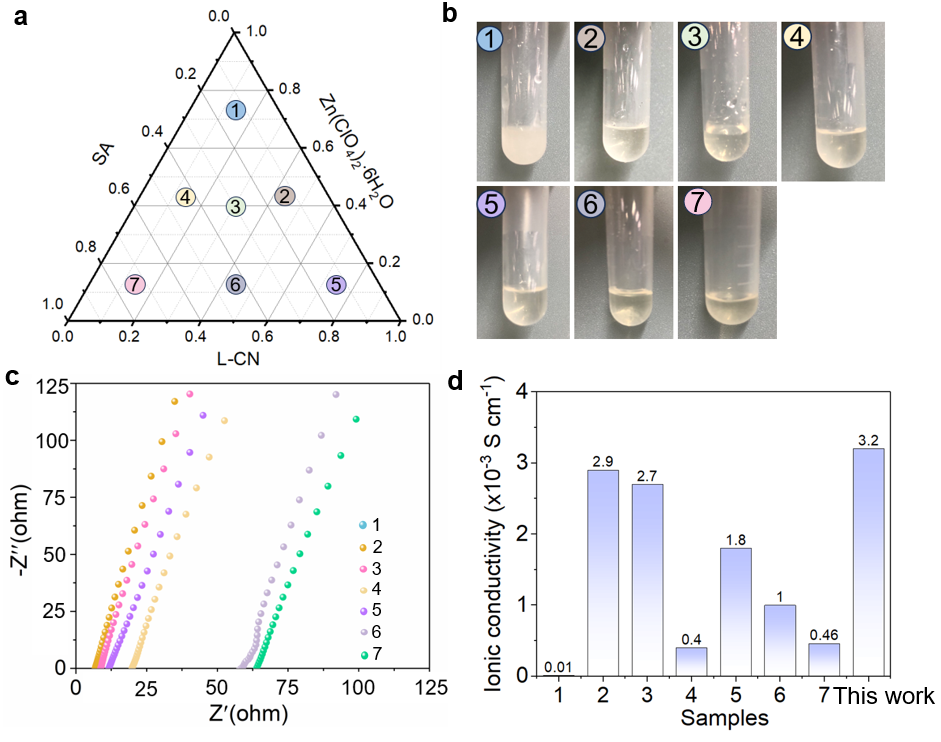


**Fig. S3** (**a**) The selected molar ratios of L-carnitine (L-CN), sulfamide (SA), and Zn(ClO_4_)_2_∙6H_2_O during screening process according to ternary phase diagram. (**b**) The corresponding physical state of the L-CN–LiClO_4_·6H_2_O–SA mixture. The AC impedance spectroscopy (**c**) and corresponding ionic conductivities (**d**) of the L-CN–LiClO_4_·6H_2_O–SA mixture at 25 ℃


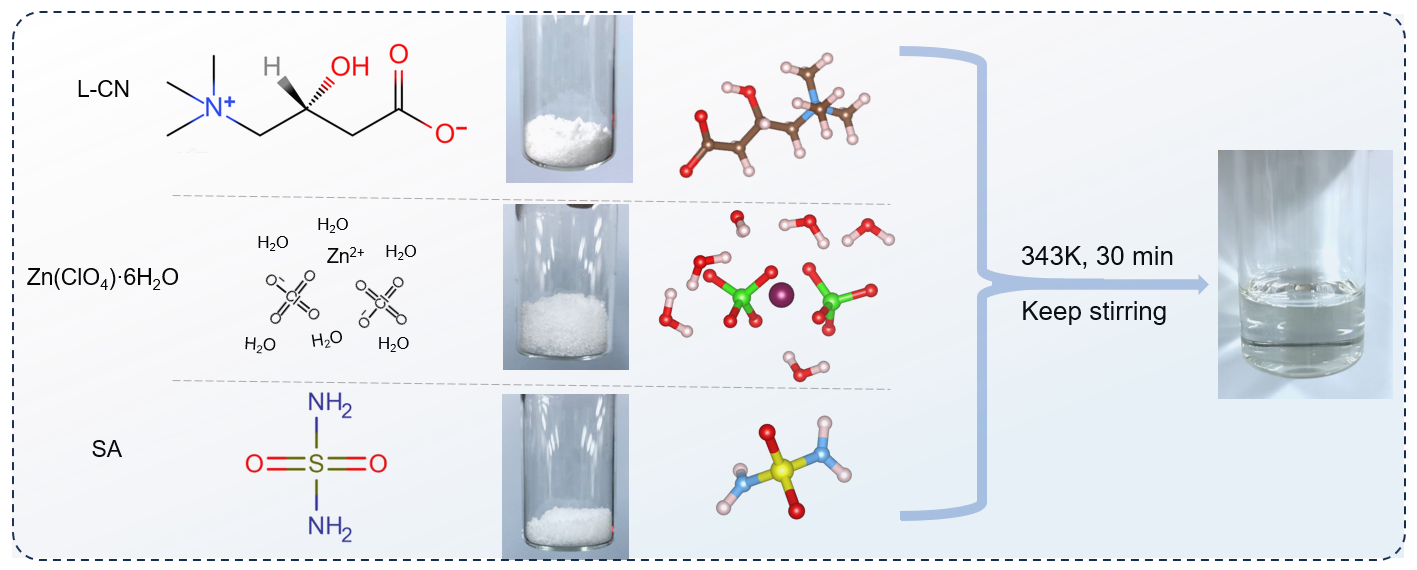


**Fig. S4** Schematic diagram of the preparation of hydrated eutectic zinc ion electrolyte

**Note:** In order to obtain optimal zinc ions ternary hydrated eutectic (ZTE) electrolyte, we studied the physicochemical properties of electrolytes derived from different molar ratios. The ternary hydrated eutectic electrolyte with the L-carnitine (L-CN), sulfamide (SA), and Zn(ClO_4_)_2_·6H_2_O in a 4:2:3 molar ratio shows low viscosity and good Zn^2+^ conductivity and phase stability, which would enable fast Zn^2+^ migration and charge transfer processes. In addition, the formed ZTE-4:3:2 electrolyte at other stoichiometric ratios easily occurs salting-out phenomenon after a period of rest. Therefore, we focused on ZTE-4:3:2 electrolyte (named ZTE) from a practical point of view in the following procedure.


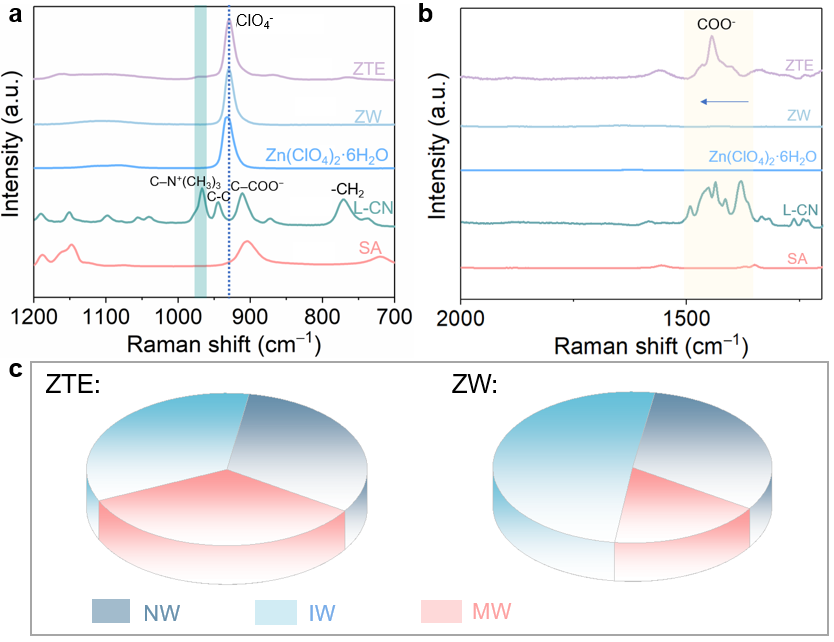


**Fig. S5** (**a-b**) Raman spectra of L-CN, Zn(ClO_4_)_2_·6H_2_O, SA, ZW, and ZTE. (**c**) Comparison of the areal ratios of network water (NW), intermediate water (IW), and multimer water (MW) in ZW and ZTE electrolytes derived from fitting accumulated peaks between 2700 and 3800 cm^−1^ in FTIR


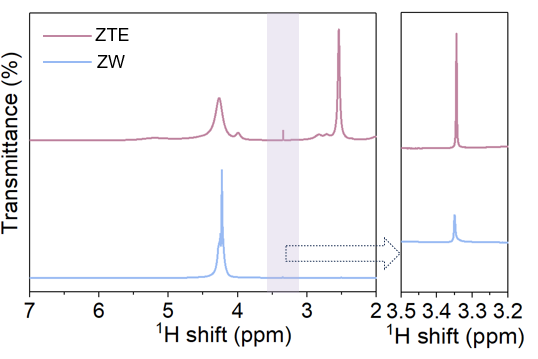


**Fig. S6** The chemical shifts for ^1^H of ZTE and ZW electrolytes

**Note**: In the ZTE electrolyte, the ^1^H peak attributed to H_2_O exhibits stronger peak intensity means the water molecules are locked in a eutectic network.


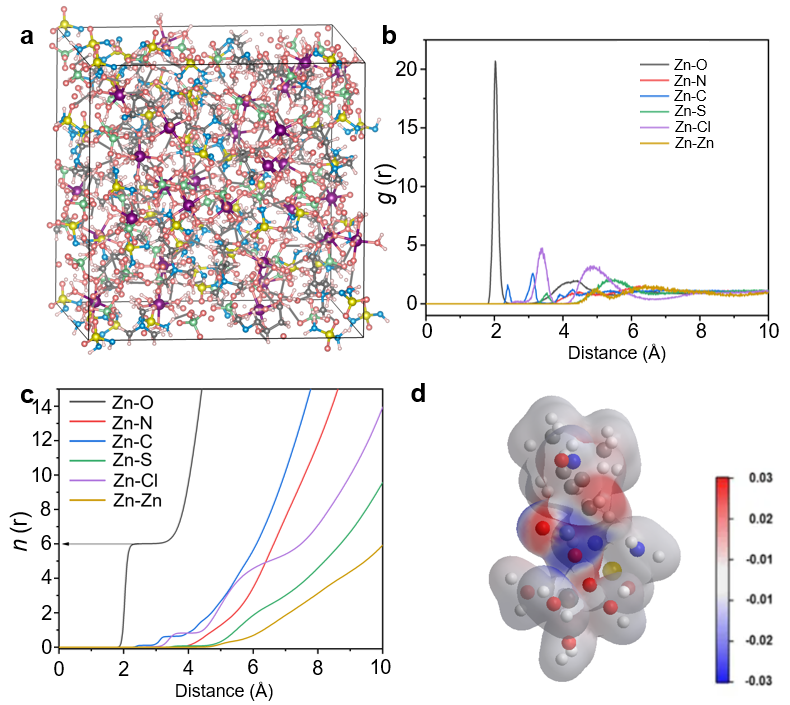


**Fig. S7** (**a**) MD snapshot of ZTE electrolyte, Zn, S, O, C, N, and H atoms are represented by dark purple, yellow, red, brown, light blue, and pink balls, respectively. (**b**) RDFs and corresponding (**c**) average coordination number of ZTE electrolyte. (**d**) MESP mappings of Zn[(L-CA)(SA)(H_2_O)_4_]^2+^ solvated complex


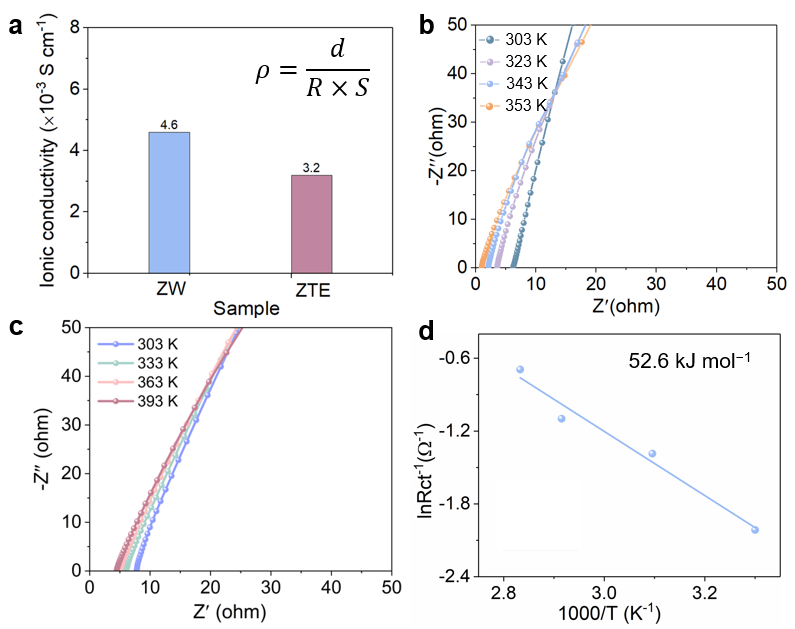


**Fig. S8** (**a**) The ion conductivity of the ZW and ZTE electrolytes at 30 ℃. The EIS spectra of the (**b**) ZW and (**c**) ZTE electrolytes at four different temperatures. (**d**) Arrhenius curves and corresponding activation energies of ZW electrolyte

**Note:** The symmetric stainless steel (SS)/ZTE/SS cell was assembled and heated to a specified temperature in an oven. EIS data were acquired using an electrochemical workstation, with frequencies ranging from 100 MHz to 0.1 Hz. For accuracy and reliability, the procedure was systematically conducted across multiple temperature ranges, starting from lower to higher temperatures.


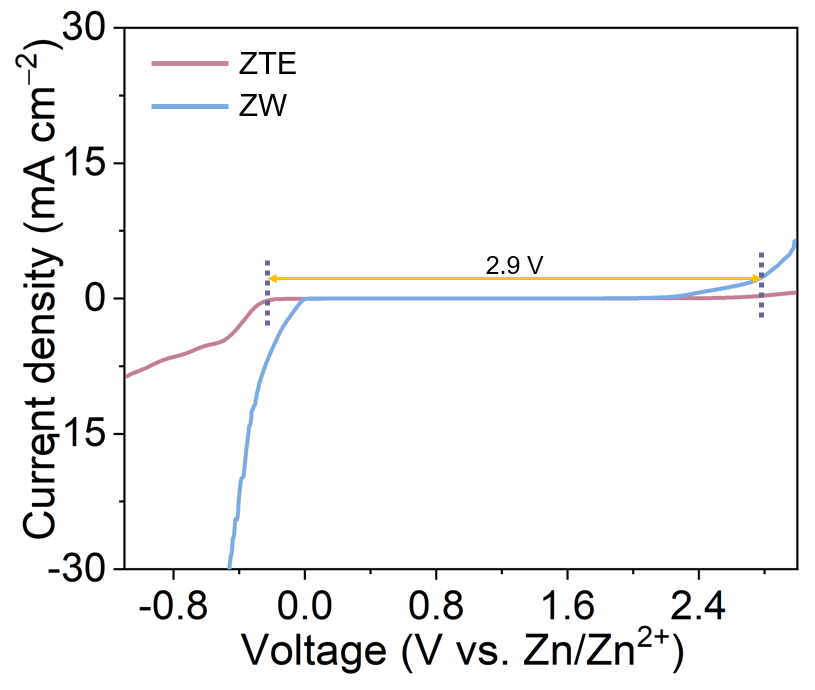


**Fig. S9** The LSV curves of the ZTE and ZW at scan rate of 0.5 mV s^-1^


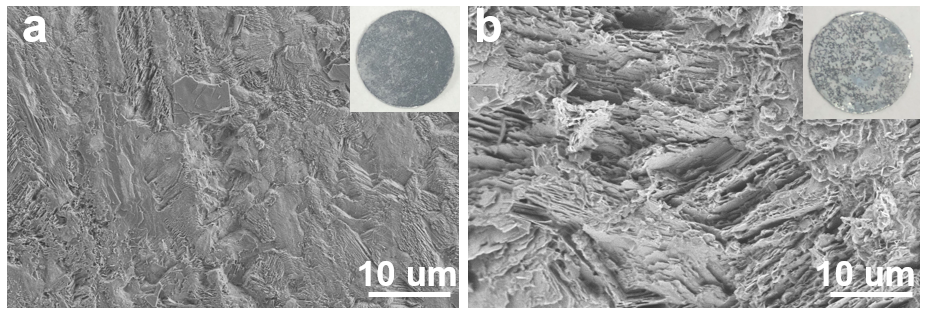


**Fig. S10** The SEM and corresponding optical images of the Zn anode after depositing for 2.0 mA h cm^−2^ in (**a**) ZTE and (**b**) ZW electrolytes, respectively


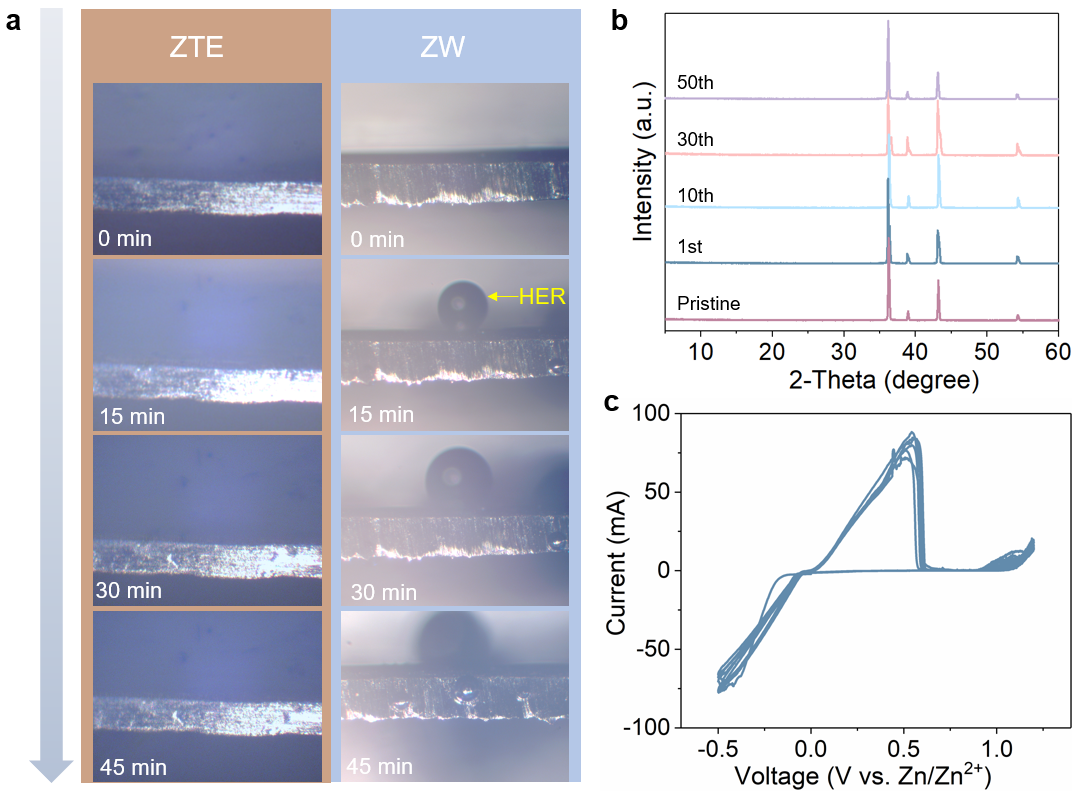


**Fig. S11** (**a**) *In situ* optical microscopy investigations of the Zn deposition using ZTE (left) and ZW (right) electrolytes. (**b**) XRD patterns of the Zn after different cycles in the ZTE electrolyte. (**c**) CV curves of Zn//Ti cells with ZW electrolyte at 0.5 mV s^−1^


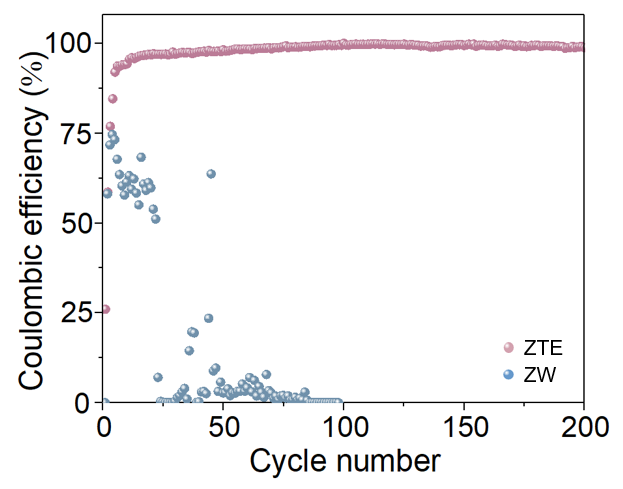


**Fig. S12** Coulombic eﬃciency comparison of Zn plating/stripping behaviors in ZW and ZTE with an area capacity of 0.5 mA h cm^−2^ (current density of 0.5 mA cm^−2^) (25 ℃)


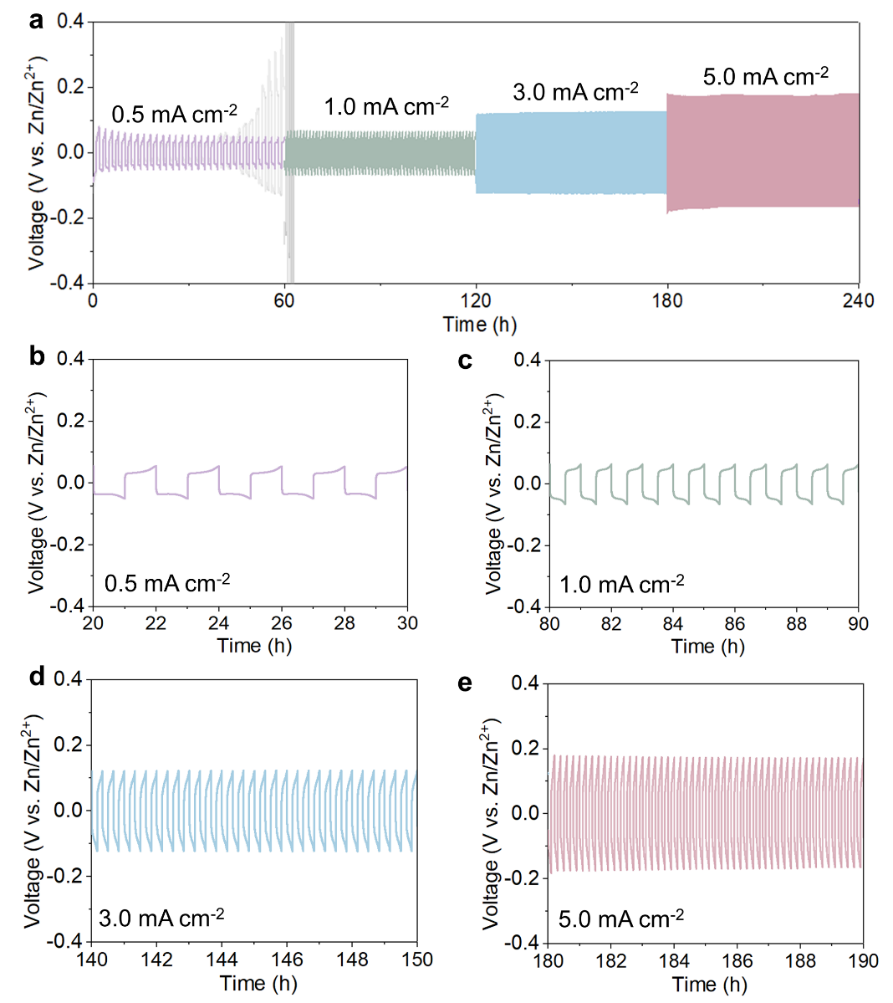


**Fig. S13** (**a**) Voltage responses of Zn/Zn cell with ZTE and ZW at rates of 0.5-5.0 mA cm^−2^ (each half cycle lasts for 0.5 mAh). (**b-e**) The enlarged partial details voltage responses of Zn//Zn symmetrical battery with ZTE

**Note:** The potential of ZTE for supporting the reversible Zn electrochemistry was also investigated under galvanostatic conditions at elevated current densities from 0.5-5.0 mA cm^−2^.


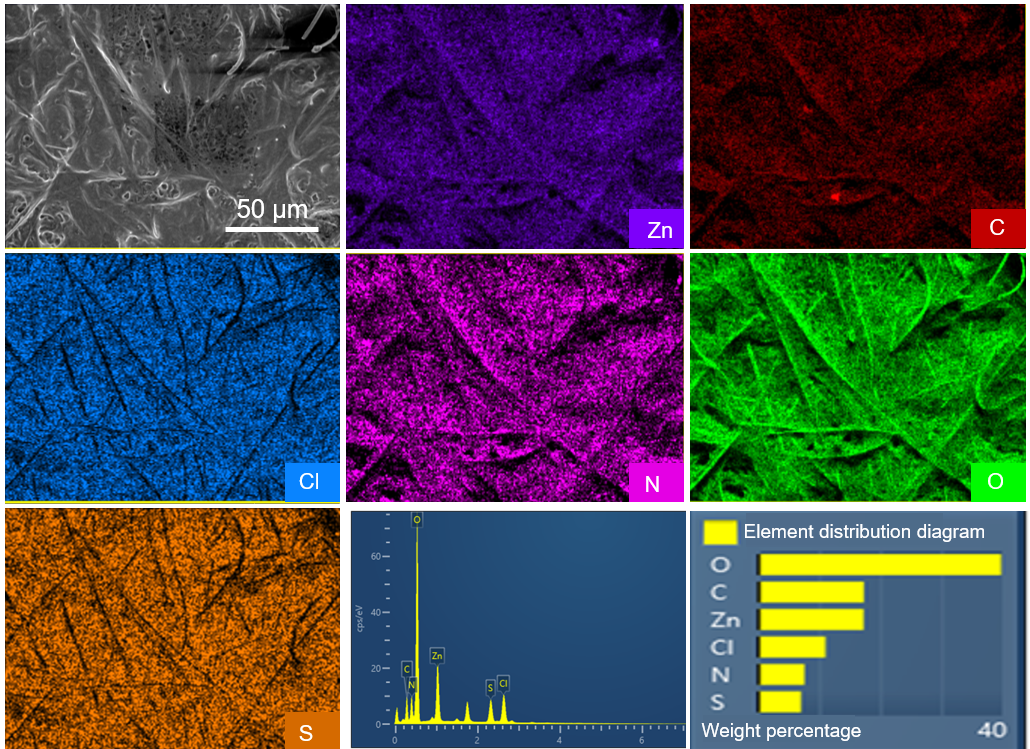


**Fig. S14** SEM image and corresponding EDS mapping of the Zn anode after platting 5.0 mAh in ZTE electrolyte


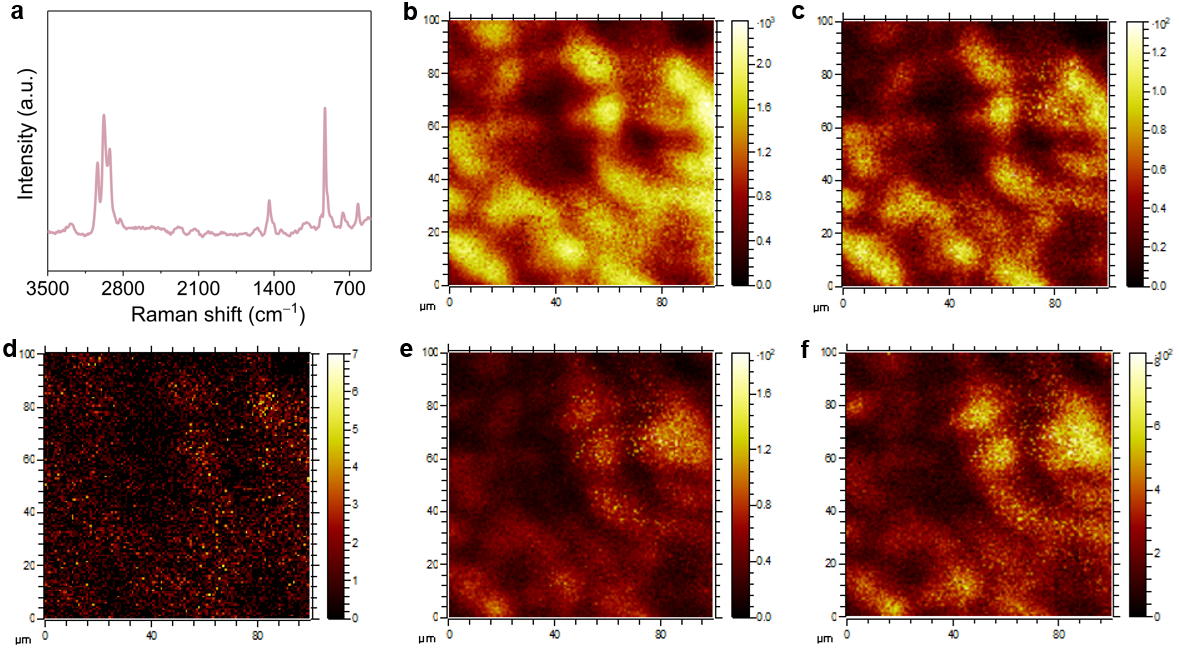


**Fig. S15** The Raman spectrum of the Zn anode after platting for 2.0 mAh cm^-2^ with ZTE electrolyte. 2D TOF-SIMS images of the (**a**) CNH_2_^−^, (**b**) ZnO^−^, (**c**) COO^−^, (**d**) CH_2_^−^, (**e**) S^2−^, and (**f**) SO_3_^−^ products of the Zn anode cycled with ZTE electrolyte


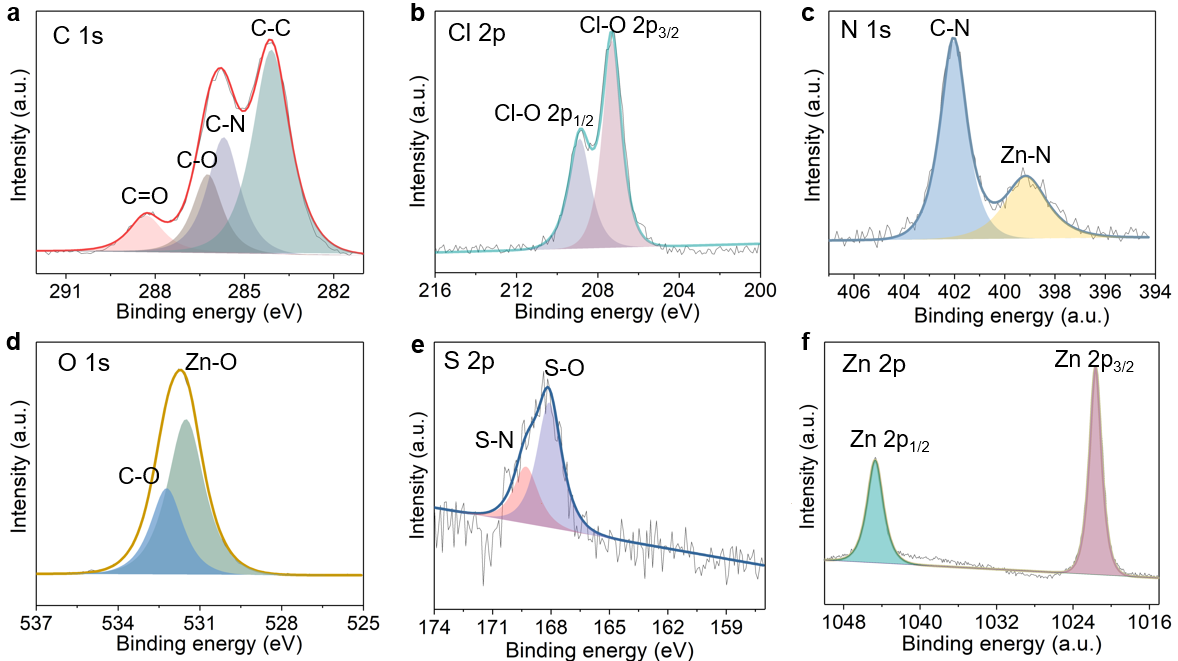


**Fig. S16** XPS spectra of (**a**) C 1s, (**b**) Cl 2p, (**c**) N 1s, (**d**) O 1s, (**e**) S 2p, and (**f**) Zn 2p of Zn anode after platting in the ZTE electrolyte


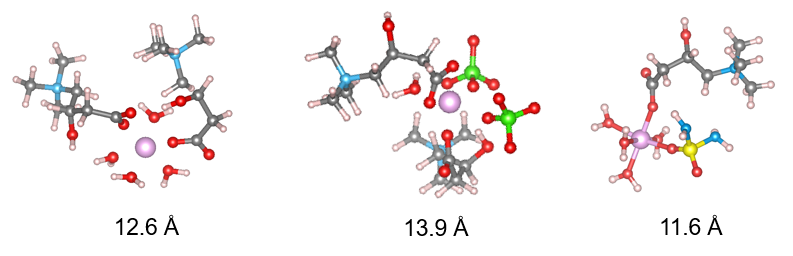


**Fig. S17** The sizes of the various of Zn^2+^ solvated clusters

**Note:** In the eutectic electrolyte, the above three solvation structures exist, but Zn[(L-CN)(SA)(H_2_O)_4_]^2+^ is the dominant one.


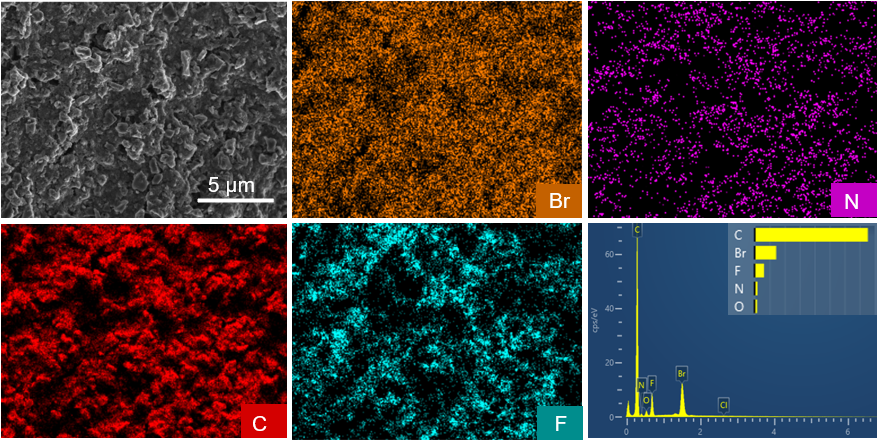


**Fig. S18** **Elemental analysis.** (**a**) SEM image and (**b, c**) element distribution maps of the prepared TBABr_3_-solid@C composite cathode. (**d**) The total spectrum of element distribution


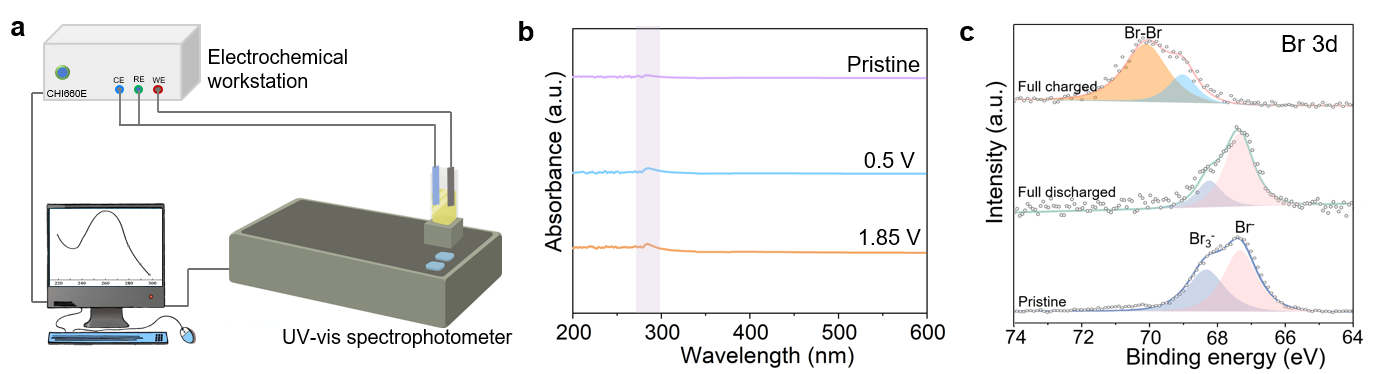


**Fig. S19** (**a**) Set-up of the *in-situ* UV-vis experiment. (**b**) UV-vis spectra of the ZTE electrolyte with Br_2_-solide electrodes at specific voltages during the charge/discharge process. (**c**) The Br 3d XPS spectra of Br_2_-solide at specific voltages in ZTE electrolyte during the charge/discharge process.

**Note:** During the charge and discharge process, no dissolution of polybrominated compounds was detected in the UV-vis spectrum, indicating that the electrolyte has satisfactory stability for bromide conversion. In addition, further analysis by X-ray photoelectron spectroscopy (XPS) spectroscopy confirmed that the bromine species exhibited good reversibility during charging and discharging.


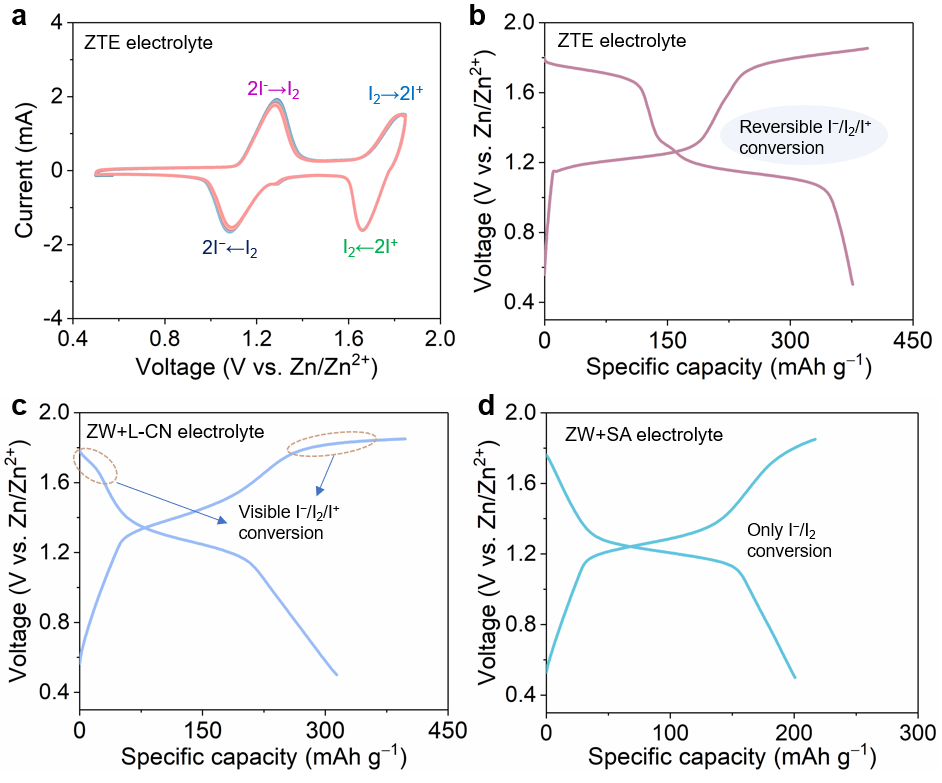


**Fig. S20** (**a**) CV curves and (**b**) voltage specific capacity curve of Zn-I_2_ battery using as I_2_@C cathode and ZTE as electrolyte. Voltage-specific capacity curve of Zn-I_2_ battery using (**c**) ZW+L-CN and (**d**) ZW+SA as electrolytes, respectively.

**Note:** The previously reported literature has confirmed that the Cl^−^ can activate the high valance oxidation of I species. Therefore, in order to figure out the pivotal role of components of the electrolyte that can stabilize the 2I^−^/I_2_/2I^+^ four-electron conversion in this work, we further conducted an electrochemistry test using ZW+L-CN and ZW+SA electrolytes, respectively. Generally, there is a lot of free water in the 2M Zn(OTf)_2_ electrolyte, which will cause hydrolysis of iodide cations. The water molecule is hydrogen bonds locked by L-CN (or SA) to further reduce its activity by adding a large number of L-CN (or SA) molecules, For the cell using the Zn(OTf)_2_/L-CN electrolyte, the CV curves demonstrate two couples of redox peaks, suggesting the I^+^ was stabilized by L-CN and realized the 2I^−^/I_2_/2I^+^ four-electron conversion in other electrolyte systems. Therefore, the above results exclude ClO_4_^−^ influence in stabilizing iodine cations.


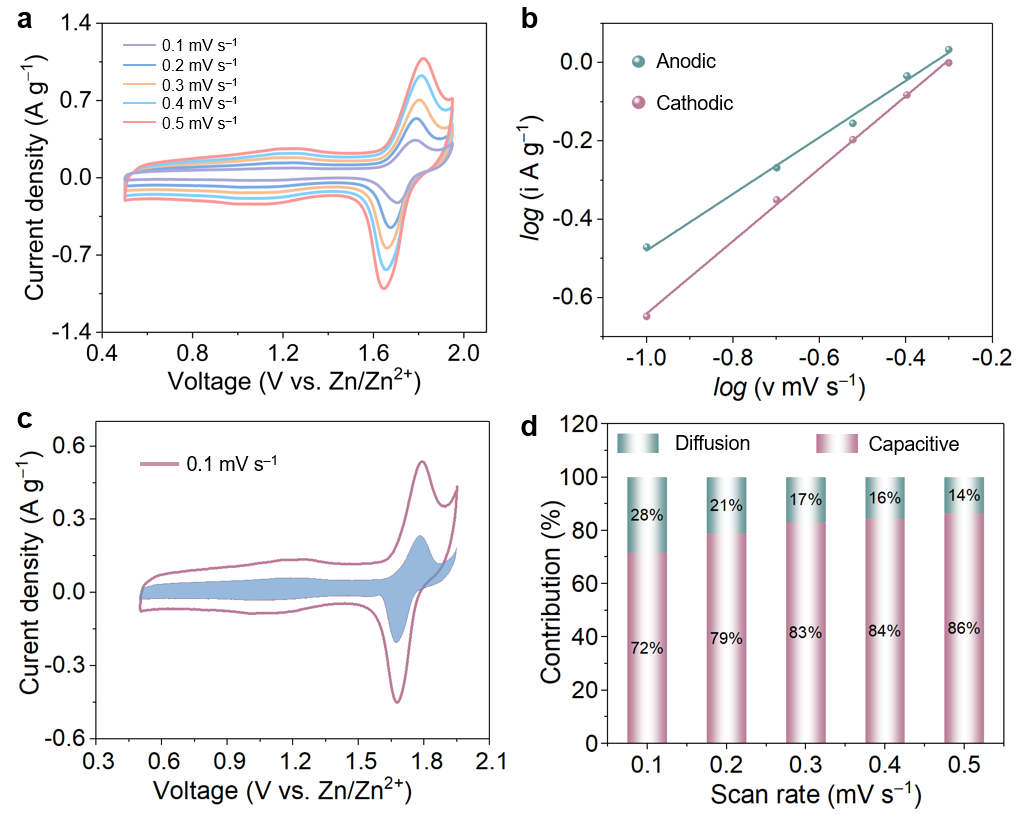


**Fig. S21** (**a**) The CV curves were obtained at different scan rates. (**b**) plots of *i* vs. *v* at sharp cathodic/anodic peak pair (peak current: *i*, scanning rate: *v*). (**c**) Capacitive contribution at 1.5 mV s^-1^. (**d**) Ratios of capacitive and diffusion-controlled contribution at various scan rates

To further understand the kinetics origin, the CV curves of the TBABr_3_@C composite cathode at various scan rates from 0.1 to 0.5 mV s^−1^ between 0.5 and 1.95 V (vs. Zn^2+^/Zn). As shown in (a), the CV curves exhibit similar peak shapes and shifts during both anodic and cathodic processes. As clearly displayed, a slightly positive shift in the anodic peaks and a negative shift in the cathodic peaks can be observed with an increasing scan rate, which is caused by the polarization eﬀect during the cycling process. The degree of the capacitive eﬀect can be qualitatively analyzed based on the relationship between the measured current (i) and scan rate (v) from the CV curves [S1]:

*i* = av^b^

where a and b are two adjustable constants. The value of b is in the range of 0.5 to 1.0, which is determined by the slope of the log(i) vs. log(v) plot. When b is close to 1.0, the electrochemical behavior is dominated by a surface capacitive process, whereas b approaches 0.5, indicating a diﬀusion-controlled process. In the current experiments, the values of b for the cathodic and anodic sweep processes are calculated to be 0.72, 0.79, 0.83, 0.84, and 0.86, respectively, suggesting the more significant capacitive storage kinetics of the TBABr_3_@C composite cathode in the ZTE electrolyte.


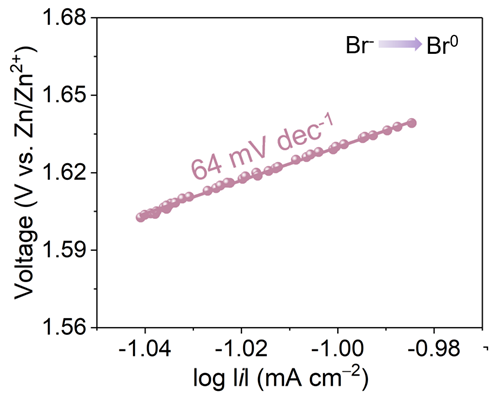


**Fig. S22** Tafel plots from CV curves in ZTE electrolytes corresponding Br_2_/Br^-^ redox process


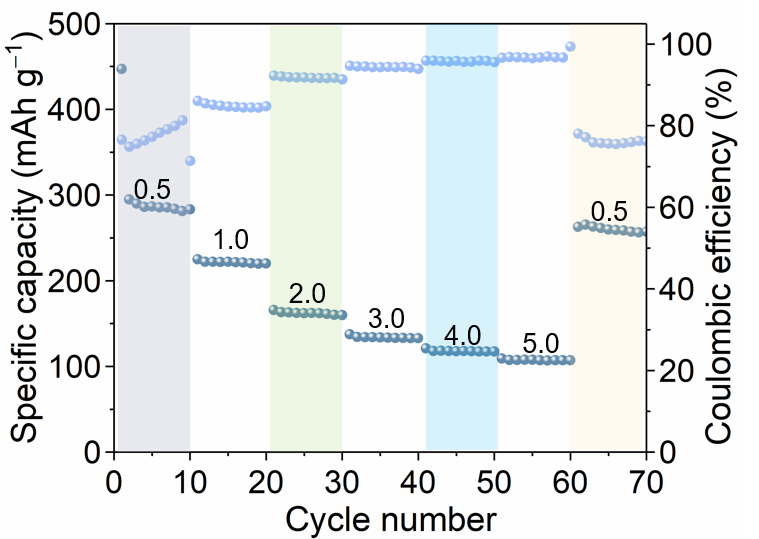


**Fig. S23** Rate performance of ZBB in ZW electrolyte (the capacity is calculated based on the active materials of Br_2_)


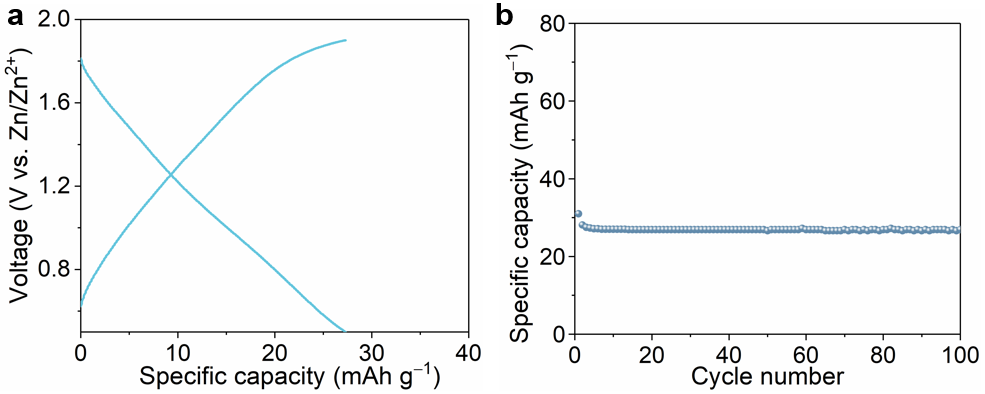


**Fig. S24** (**a**) charge-discharge curves and (**b**) cycling performance of carbon cloth electrode at 0.5 A g^-1^


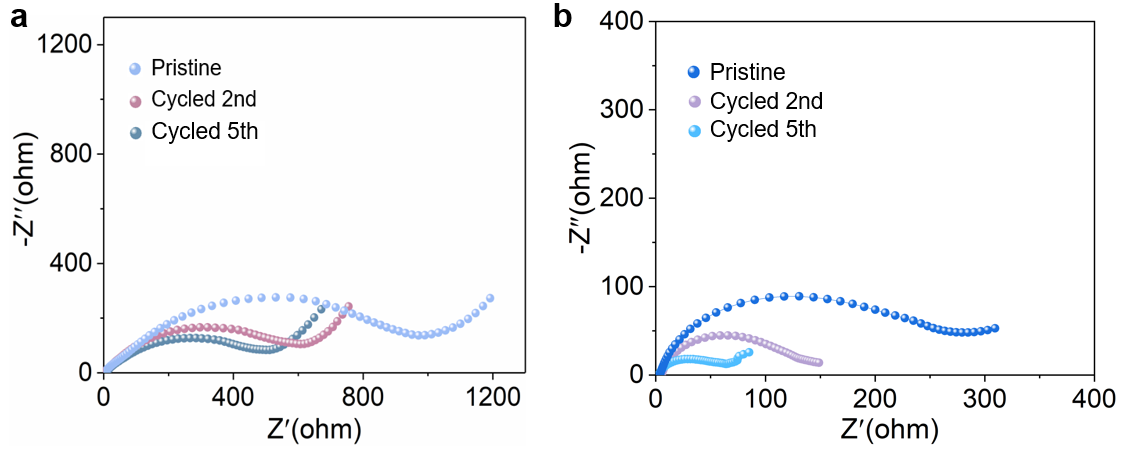


**Fig. S25** The EIS spectra of Zn-Br_2_ batteries that have undergone cycling in (**a**) ZTE and (**b**) ZW electrolytes at diferent cycles

**Table S1** log P value of the L-CN, SA, H_2_O and ClO_4_^-^ species

| Species | L-CN | SA | H_2_O | ClO_4_^-^ |
| --- | --- | --- | --- | --- |
| log P | -5.5 | -1.52 | -0.29 | -2.58 |

**Note:** logP = log([solute in octanol]/[solute inwater]), describes how the concentration of a solute partitions in an octanol and water two-phase system.

**Table S2** Comparison of electrochemical performances of this work to reported halogen-Zn, Mn-Zn, and V-Zn battery systems

| Cathodes | | Electrolyte | Anode | Specific capacity | Refs. |
| --- | --- | --- | --- | --- | --- |
| halogen-Zn | Br_2_-solid@C | ZTE | Zn | 283.4 mAh g^-1^ at 0.5 A g^-1^ | This work |
|  | KBr-NiPPc | 2M ZnSO_4_ | Zn | 285 mAh g^-1^ at 1.0 A g^-1^ | [S2] |
|  | Br_2_-solid@C | 2M ZnSO_4_(IU)_0.25_ | Zn | 264.1 mAh g^-1^ at 0.5 A g^-1^ | [S3] |
|  | I_2_@PAC | 1M ZnSO_4_ | Zn | 220 mAh g^-1^ at 0.5 C | [S4] |
|  | I_2_-ZIF | 0.5M ZnSO_4_ | Zn | 182 mAh g^-1^ at 0.2 A g^-1^ | [S5] |
|  | ZnI_2_@KB | 2M ZnSO_4_ | Zn | 287 mAh g^-1^ at 0.1 A g^-1^ | [S6] |
|  | Co_9_S_8_-NC/I_2_ | 7.5 m ZnCl_2_ | Zn | 365 mAh g^-1^ at 1.0 A g^-1^ | [S7] |
| Zn-Mn | γ-MnO_2_ | 1M ZnSO_4_ | Zn | 285 mAh g^-1^ at 0.05 A g^-1^ | [S8] |
|  | β-MnO_2_ | 1M ZnSO_4_ | Zn | 270 mAh g^-1^ at 0.1 A g^-1^ | [S9] |
|  | MnO_2_ | 1M ZnSO_4_  +0.2M MnSO_4_ | Zn | 290 mAh g^-1^ at 0.1 A g^-1^ | [S10] |
|  | MnO_2_ | ZSC-gel | Zn | 275 mAh g^-1^ at 0.3 A g^-1^ | [S11] |
|  | γ-MnO_2_ | Zn(CF_3_SO_3_)_2_  +Mn(CF_3_SO_3_)_2_ | Zn | 132 mAh g^-1^ at 0.15 A g^-1^ | [S12] |
|  | Mn_3_O_4_ | 2M ZnSO_4_ | Zn | 239 mAh g^-1^ at 0.1 A g^-1^ | [S13] |
|  | δ-MnO_2_ | 2M ZnSO_4_  +0.2M MnSO_4_ | Zn | 278 mAh g^-1^ at 0.3 A g^-1^ | [S14] |
| Zn-V | Zn_0.25_V_2_O_5_·nH_2_O | 1M ZnSO_4_ | Zn | 260 mAh g^-1^ at 0.3 A g^-1^ | [S15] |
|  | Ca_0.24_V_2_O_5_·0.83H_2_O | 1M ZnSO_4_ | Zn | 180 mAh g ^-1^ at 0.5 A g^-1^ | [S16] |
|  | V_2_O_5_ | BE + CAT | Zn | 305 mAh g ^-1^ at 1.0 A g^-1^ | [S17] |
|  | V_2_O_5_·nH_2_O | 3M Zn(CF_3_SO_3_)_2_ | Zn | 381 mAh g ^-1^ at 0.06 A g^-1^ | [S18] |
|  | H_2_V_3_O_8_ | 3M Zn(CF_3_SO_3_)_2_ | Zn | 240 mAh g ^-1^ at 1 A g^-1^ | [S19] |
|  | VO_2_ | Me56 | Zn | 311 mAh g ^-1^ at 0.5 A g^-1^ | [S20] |
|  | V_2_O_5_/V_3_O_7_·nH_2_O | Zn(CF_3_SO_3_)_2_  + LiTFSI | Zn | 250 mAh g ^-1^ at 0.5 A g^-1^ | [S21] |
| Zn-Organic | HAQ-COF | 2 M ZnSO_4_ | Zn | 339 mAh g ^-1^ at 0.1 A g^-1^ | [S22] |
|  | PQ-MCT | 1Zn-0.5Sc in  AcNDI_4_ | Zn | 120.8 mAh g ^-1^ at 0.1 A g^-1^ | [S23] |
|  | PANI | ZnCl_2_/EG | Zn | 180 mAh g ^-1^ at 2 A g^-1^ | [S24] |
|  | PTD-1 | 2 M ZnSO_4_ | Zn | 145 mAh g ^-1^ at 0.1 A g^-1^ | [S25] |
|  | PQ-MCT | 0.5m ZnTFMS/DMF | Zn | 131 mAh g ^-1^ at 0.2 A g^-1^ | [S26] |
|  | PDBS | PAM hydrogel | Zn | 220 mAh g ^-1^ at 0.1 A g^-1^ | [S27] |
|  | HATN-PNZ | 3M Zn(CF_3_SO_3_)_2_ | Zn | 257 mAh g ^-1^ at 5 A g^-1^ | [S28] |
|  | PANI | 2M ZnSO_4_ + 0.01 m SG | Zn | 192 mAh g ^-1^ at 1 A g^-1^ | [S29] |

**Calculations:**

The theoretical capacity of Br_2_-solid@C cathode based two-electron conversion is:

*C*$=\frac{nF}{M}=\frac{2\times26800\frac{mAh}{mol}}{2\times79.9\frac{g}{mol}}=$335.4$\frac{\mathrm{mAh}}{g}$

The electromotive force of Zn-Br_2_ battery is:

*V*$=-\frac{\Delta Gf}{nF}=\frac{346.5\frac{kJ}{mol}}{2\times94,685\frac{C}{mol}}=$1.83 V

The theoretical energy density of Br_2_ cathode based two-electron conversion is:

*E=C*$\times V=335.4\frac{\mathrm{mAh}}{g}\times1.83 V=$613 Wh/kg

The theoretical energy density of Zn-Br_2_ battery based two-electron conversion is:

*E*$=-\frac{\Delta Gf}{M}=\frac{346.5\frac{kJ}{mol}}{225.2\frac{g}{mol}}=$1538$\frac{\mathrm{kJ}}{\mathrm{kg}}=$427$\frac{\mathrm{Wh}}{\mathrm{kg}}$

**Supplementary References**

1. T. Wei, Q. Li, G. Yang, C. Wang, Pseudo-Zn–air and Zn-ion intercalation dual mechanisms to realize high-areal capacitance and long-life energy storage in aqueous Zn battery. Adv. Energy Mater. **9**, 1901480 (2019). <https://doi.org/10.1002/aenm.201901480>
2. H. Wei, G. Qu, X. Zhang, B. Ren, S. Li et al., Boosting aqueous non-flow zinc–bromine batteries with a two-dimensional metal–organic framework host: an adsorption-catalysis approach. Energy Environ. Sci. **16**, 4073–4083 (2023). <https://doi.org/10.1039/D3EE01639K>
3. X. Wang, Y. Ying, X. Li, S. Chen, G. Gao et al., Preferred planar crystal growth and uniform solid electrolyte interfaces enabled by anion receptors for stable aqueous Zn batteries. Energy Environ. Sci. **16**, 4572–4583 (2023). <https://doi.org/10.1039/D3EE01580G>
4. P. Yang, K. Zhang, S. Liu, W. Zhuang, Z. Shao et al., Ionic selective separator design enables long-life zinc–iodine batteries *via* synergistic anode stabilization and polyiodide shuttle suppression. Adv. Funct. Mater., 2410712 (2024). <https://doi.org/10.1002/adfm.202410712>
5. Z. Wang, J. Huang, Z. Guo, X. Dong, Y. Liu et al., A metal-organic framework host for highly reversible dendrite-free zinc metal anodes. Joule **3**, 1289–1300 (2019). <https://doi.org/10.1016/j.joule.2019.02.012>
6. Q. Chen, J. Hao, Y. Zhu, S.-J. Zhang, P. Zuo et al., Anti-swelling microporous membrane for high-capacity and long-life Zn-I_2_ batteries. Angew. Chem. Int. Ed. e202413703 (2024). <https://doi.org/10.1002/anie.202413703>
7. T. Hu, Y. Zhao, Y. Yang, H. Lv, R. Zhong et al., Development of inverse-opal-structured charge-deficient Co_9_S_8_@nitrogen-doped-carbon to catalytically enable high energy and high power for the two-electron transfer I^+^/I^-^ electrode. Adv. Mater. **36**, e2312246 (2024). <https://doi.org/10.1002/adma.202312246>
8. M.H. Alfaruqi, V. Mathew, J. Gim, S. Kim, J. Song et al., Electrochemically induced structural transformation in a γ-MnO_2_ cathode of a high capacity zinc-ion battery system. Chem. Mater. **27**, 3609–3620 (2015). <https://doi.org/10.1021/cm504717p>
9. S. Islam, M.H. Alfaruqi, V. Mathew, J. Song, S. Kim et al., Facile synthesis and the exploration of the zinc storage mechanism of β-MnO_2_ nanorods with exposed (101) planes as a novel cathode material for high performance eco-friendly zinc-ion batteries. J. Mater. Chem. A **5**, 23299–23309 (2017). <https://doi.org/10.1039/C7TA07170A>
10. W. Sun, F. Wang, S. Hou, C. Yang, X. Fan et al., Zn/MnO_2_ battery chemistry with H^+^ and Zn^2+^ coinsertion. J. Am. Chem. Soc. **139**, 9775–9778 (2017). <https://doi.org/10.1021/jacs.7b04471>
11. F. Mo, Z. Chen, G. Liang, D. Wang, Y. Zhao et al., Zwitterionic sulfobetaine hydrogel electrolyte building separated positive/negative ion migration channels for aqueous Zn-MnO_2_ batteries with superior rate capabilities. Adv. Energy Mater. **10**, 2000035 (2020). <https://doi.org/10.1002/aenm.202000035>
12. N. Zhang, F. Cheng, J. Liu, L. Wang, X. Long et al., Rechargeable aqueous zinc-manganese dioxide batteries with high energy and power densities. Nat. Commun. **8**, 405 (2017). <https://doi.org/10.1038/s41467-017-00467-x>
13. J. Hao, J. Mou, J. Zhang, L. Dong, W. Liu et al., Electrochemically induced spinel-layered phase transition of Mn_3_O_4_ in high performance neutral aqueous rechargeable zinc battery. Electrochim. Acta **259**, 170–178 (2018). <https://doi.org/10.1016/j.electacta.2017.10.166>
14. D. Wang, L. Wang, G. Liang, H. Li, Z. Liu et al., A superior δ-MnO_2_ cathode and a self-healing Zn-δ-MnO_2_ battery. ACS Nano **13**, 10643–10652 (2019). <https://doi.org/10.1021/acsnano.9b04916>
15. D. Kundu, B.D. Adams, V. Duffort, S.H. Vajargah, L. Nazar, A high-capacity and long-life aqueous rechargeable zinc battery using a metal oxide intercalation cathode. Nat. Energy **2016**, 1*,* 1-8. <https://doi.org/10.1038/nenergy.2016.119>
16. Q. Yang, X. Qu, H. Cui, X. He, Y. Shao et al., Rechargeable aqueous Mn-metal battery enabled by inorganic-organic interfaces. Angew. Chem. Int. Ed **61**, e202206471 (2022). <https://doi.org/10.1002/anie.202206471>
17. F. Zhang, T. Liao, H. Peng, S. Xi, D.-C. Qi et al., Outer sphere electron transfer enabling high-voltage aqueous electrolytes. J. Am. Chem. Soc. **146**, 10812–10821 (2024). <https://doi.org/10.1021/jacs.4c01188>
18. M. Yan, P. He, Y. Chen, S. Wang, Q. Wei et al., Water-lubricated intercalation in V_2_ O_5_ ·nH_2_ O for high-capacity and high-rate aqueous rechargeable zinc batteries. Adv. Mater. **30**, 1703725 (2018). <https://doi.org/10.1002/adma.201703725>
19. P. He, Y. Quan, X. Xu, M. Yan, W. Yang et al., High-performance aqueous zinc–ion battery based on layered H_2_V_3_O_8_ nanowire cathode. Small **13**, 1702551 (2017). <https://doi.org/10.1002/smll.201702551>
20. W. Xu, J. Li, X. Liao, L. Zhang, X. Zhang et al., Fluoride-rich, organic-inorganic gradient interphase enabled by sacrificial solvation shells for reversible zinc metal batteries. J. Am. Chem. Soc. **145**, 22456–22465 (2023). <https://doi.org/10.1021/jacs.3c06523>
21. X. Li, L. Ma, Y. Zhao, Q. Yang, D. Wang et al., Hydrated hybrid vanadium oxide nanowires as the superior cathode for aqueous Zn battery. Mater. Today Energy **14**, 100361 (2019). <https://doi.org/10.1016/j.mtener.2019.100361>
22. W. Wang, V.S. Kale, Z. Cao, Y. Lei, S. Kandambeth et al., Molecular engineering of covalent organic framework cathodes for enhanced zinc-ion batteries. Adv. Mater. **33**, e2103617 (2021). <https://doi.org/10.1002/adma.202103617>
23. M. Kim, S.J. Shin, J. Lee, Y. Park, Y. Kim et al., Cationic additive with a rigid solvation shell for high-performance zinc ion batteries. Angew. Chem. Int. Ed. **61**, e202211589 (2022). <https://doi.org/10.1002/anie.202211589>
24. L. Geng, J. Meng, X. Wang, C. Han, K. Han et al., Eutectic electrolyte with unique solvation structure for high-performance zinc-ion batteries. Angew. Chem. Int. Ed **61**, e202206717 (2022). <https://doi.org/10.1002/anie.202206717>
25. N. Wang, Z. Guo, Z. Ni, J. Xu, X. Qiu et al., Molecular tailoring of an n/p-type phenothiazine organic scaffold for zinc batteries. Angew. Chem. Int. Ed. **60**, 20826–20832 (2021). <https://doi.org/10.1002/anie.202106238>
26. J. Chen, Y. Peng, Y. Yin, Z. Fang, Y. Cao et al., A desolvation-free sodium dual-ion chemistry for high power density and extremely low temperature. Angew. Chem. Int. Ed **60**, 23858–23862 (2021). <https://doi.org/10.1002/anie.202110501>
27. T. Sun, Z.-J. Li, Y.-F. Zhi, Y.-J. Huang, H.J. Fan et al., Poly(2, 5-dihydroxy-1, 4-benzoquinonyl sulfide) As an efficient cathode for high-performance aqueous zinc–organic batteries. Adv. Funct. Mater. **31**, 2010049 (2021). <https://doi.org/10.1002/adfm.202010049>
28. S. Li, J. Shang, M. Li, M. Xu, F. Zeng et al., Design and synthesis of a π-conjugated N-heteroaromatic material for aqueous zinc-organic batteries with ultrahigh rate and extremely long life. Adv. Mater. **35**, e2207115 (2023). <https://doi.org/10.1002/adma.202207115>
29. J. Hao, L. Yuan, Y. Zhu, M. Jaroniec, S.-Z. Qiao, Triple-function electrolyte regulation toward advanced aqueous Zn-ion batteries. Adv. Mater. **34**, e2206963 (2022). <https://doi.org/10.1002/adma.202206963>
